# Financial spillovers, spillbacks, and the scope for international macroprudential policy coordination

**DOI:** 10.1007/s10368-021-00522-5

**Published:** 2021-10-14

**Authors:** Pierre-Richard Agénor, Luiz A. Pereira da Silva

**Affiliations:** 1 School of Social Sciences, University of Manchester, Manchester, UK; 2 Bank for International Settlements, Basel, Switzerland

## Abstract

This paper discusses the scope for international macroprudential policy coordination in a financially integrated world economy. It begins with a review of the transmission channels associated with, and the empirical evidence on, financial spillovers and spillbacks. Limitations of the existing literature are also identified. The potential gains associated with cross-border macroprudential coordination, dwelling on both recent analytical contributions and quantitative studies based on multi-country models with financial frictions, are then evaluated. The issue of whether coordination of macroprudential policies simultaneously requires some degree of monetary policy coordination is also discussed. The analysis focuses on the potential for policy coordination between major advanced economies and a group identified as *systemically-important middle-income countries* (SMICs). Next, practical ways to promote international macroprudential policy coordination are considered. Following a discussion of Basel III’s Principle of reciprocity and ways to improve it, the paper advocates a further strengthening of the current statistical, empirical and analytical work conducted by international financial institutions to evaluate, and raise awareness of, the gains from international coordination of macroprudential policies.

## Introduction

**Fig. 1:**
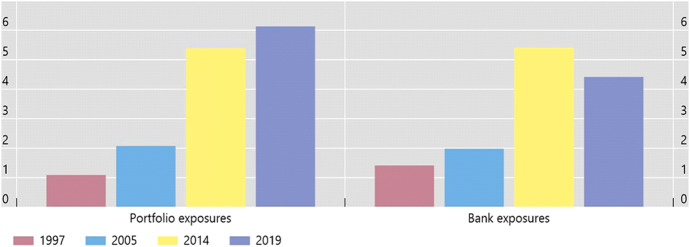
Financial Exposures of Advanced Economies to Selected Middle-Income Countries^1^. (As a percentage of GDP)^2 1^Advanced economies (AEs): Australia, Canada, Denmark, euro area, Hong Kong SAR, Israel, Japan, Norway, Singapore, Sweden, Switzerland, the United Kingdom, and the United States. Middle-income countries: Brazil, China, India, Indonesia, Mexico, Russia, South Africa and Turkey. ^2^As a percentage of advanced economies’ GDP. Sources: IMF, *Coordinated Portfolio Investment Survey*; BIS consolidated international banking statistics; BIS locational international banking statistics; BIS calculations

Over the past three decades, and despite a slowdown coinciding with the global financial crisis (GFC) of 2007–09, the degree of international financial integration has increased at a rapid pace. Changes in gross capital flows (including cross-border bank claims), gross foreign assets and liabilities, or net international asset positions, capture this process fairly well. Figure 1, for instance, shows the evolution of advanced economies’ financial exposures to a group of large middle-income countries, split into portfolio exposures and bank exposures. It shows that both types of exposures have increased substantially since the late 1990s. The rapid pace of financial globalization over the past decades has also been reflected in an over six-fold increase in the external assets and liabilities of nations as a share of GDP – despite a marked slowdown in the growth of cross-border positions in the immediate aftermath of the GFC and, more recently, disruptions to the world economy associated with the ongoing COVID-19 pandemic.^1^

Despite significant potential benefits (in terms of improved efficiency in resource allocation, for instance) financial integration and increased global interconnectedness have led to new policy challenges, associated with the amplification of shocks during turbulent times and the transmission of excess financial volatility through international capital flows.^2^ Indeed, there is robust evidence that private capital flows have been a major conduit of global financial shocks across countries and have helped fuel domestic credit booms that have often ended in financial crises, especially in developing economies. But international capital flows have created macroeconomic policy challenges for advanced economies as well. In particular, it has been argued that the rest of the world’s appetite for US safe assets was an important factor behind the credit and asset price booms in the United States that fueled the subsequent financial crisis and created turmoil around the world. It is also well documented that, since the GFC, the various forms of accommodative monetary policy pursued in the United States and the euro area have exerted significant spillover effects on other countries by influencing interest rates and credit conditions around the world – irrespective, at first sight, of the nature of the exchange rate regime.^3^ In response, many countries chose not to allow their currency to float freely to insulate themselves (as textbook discussions would suggest) and used instead a combination of sterilized intervention and capital controls – in effect, retreating from open capital markets, if only temporarily.^4^

At the same time, there is evidence to suggest that in recent years financial market volatility in some large middle-income countries has been transmitted back, and to a greater extent, to asset prices in advanced economies and other countries. For instance, the suspension of trading after the Chinese stock market drop on January 6, 2016 affected major asset markets all over the world.^5^ Thus, international spillovers have become a two-way street – with the potential to create financial instability in both directions.

International spillovers, especially those associated with monetary policy in advanced economies, can be a source of concern for another reason. Even if monetary policy is optimally tailored to macroeconomic and financial conditions in the United States or the euro area – in the sense of being geared at promoting price and output stability domestically – recipient countries may face precarious initial conditions, such as strong inflationary pressures and significant risks to financial stability (Pereira da Silva (2013)). In a context where cyclical positions are not well synchronized, international monetary policy spillovers from advanced economies could well be destabilizing for the global economy. This has led observers and policymakers in several major middle-income countries (especially Brazil and India) to issue pleas for increased policy coordination. The argument, as it is usually presented (see, for instance, Mishra and Rajan (2016) and Shin (2015)), is that policymakers in advanced economies must go beyond their national mandate – which requires taking account of the external impact of their policies only insofar as they feed back onto their own economies, through *spillback effects* – and explicitly account for the cross-border effects of their policy decisions when they act independently.

At the same time, in both advanced and developing economies, there has been greater reliance in recent years on national macroprudential policies, in both their structural and countercyclical dimensions.^6^ As documented in a number of studies, including Akinci and Olmstead-Rumsey (2018) and Borio et al. (2021), these policies (especially those of a time-varying nature) have been fairly effective in terms of improving the resilience of national financial systems. They also appear to have helped, at least to some degree, recipient countries to insulate themselves from global financial shocks and mitigate the systemic financial risks that international capital flows may create (see Ghosh et al. (2017)). Moreover, in response to these shocks, there have also been calls for greater coordination of these policies across countries.

The purpose of this paper is to discuss, from both an analytical and policy perspective, the role of, and scope for, international macroprudential policy coordination in a financially integrated world economy. Among the issues we address are the extent to which greater coordination of macroprudential policies may help to mitigate the effects of cross-border financial spillovers and spillbacks; the magnitude of potential gains from international coordination; and the role supranational authorities may, or should, play in monitoring system-wide financial risks and promoting international coordination in the area of macroprudential regulation.

At the outset, it is important to note that even though cross-border spillovers and spillbacks may be significant, and may indeed have increased in magnitude in recent years, it does not necessarily follow that they reduce global welfare and that cooperation is prima facie welfare improving. If, for instance, the global economy is experiencing a recession, the coordinated adoption of an expansionary fiscal policy stance by a group of large countries may, through trade and financial spillovers, benefit all countries. The magnitude of this gain may actually increase with the degree to which countries are interconnected, the degree of business cycle synchronization, and the very magnitude of spillovers. Alternatively, if a country becomes more resilient to global financial shocks as a result of more active macroprudential regulation, other countries may also benefit from greater stability through less volatile trade and financial flows with that country.

However, it is also possible that national policies themselves may generate greater volatility across countries, through abrupt changes in short-term capital flows induced by fluctuations in asset prices and relative rates of return. If maintaining financial stability is a key policy objective, the propagation of financial risks through these flows may become a source of concern. These risks – which may be magnified by domestic financial market imperfections – may or may not materialize in the same manner across countries, even when they are highly integrated, because short-term capital flows are not always driven by fundamentals or because countries can be at different stages of their business and financial cycles. But to the extent that financial risks represent negative externalities that tend to increase with the magnitude of spillovers and spillbacks, which may in turn be exacerbated (through cross-country leakages) by uncoordinated national macroprudential policies, there is a case for macroprudential policy coordination.^7^

We focus our analysis on major advanced economies and a group of countries that we identify as *systemically-important middle-income countries* (SMICs), rather than “emerging markets” – a term that, in our view, has become largely obsolete.^8^ Specifically, we identify this group as consisting of eight countries: Brazil, China, India, Indonesia, Mexico, Russia, South Africa, and Turkey.^9^ Undoubtedly, these countries differ significantly in terms of a number of “real” structural characteristics (population size, shares of savings and investment in GDP, trade composition, and so on), the nature of their exchange rate regime, and their degree of international financial integration –with China and India being significantly less financially open than the others. However, they are also relatively homogeneous in terms of the type of frictions and imperfections that characterize their financial systems, their inability to borrow in their own currency, their vulnerability to global shocks and adverse tail events, their importance for commodity markets (both as suppliers and demanders), and the spillbacks that they can potentially generate for advanced economies – either in the recent past or in the foreseeable future.

Indeed, although SMICs remain predominantly a destination, rather than a source, of global financial spillovers, the main conduit for these spillovers (capital flows) can cause a gradual accumulation of imbalances that can later result in substantial spillbacks to advanced economies (Bank for International Settlements (2016)). The fact that SMICs account for a growing share of both world GDP (from 10.6% in the late 1990s to 18.9% in 2011–15) and world exports of goods and services (from about 10% in the late 1990s to 20% in 2011–15) also creates the possibility of a trade channel through which spillback effects may occur. These features are important from the perspective of this study. Moreover, these countries have been *statistically* identified by the International Monetary Fund (2016a, Chapter 2; 2016c)) as generating significant spillback effects on advanced economies in recent years, especially through equity markets. Our premise therefore is that they stand to benefit the most from international coordination with major advanced economies, and vice versa. More generally, our view is that promoting global macroprudential policy coordination, especially at the high-frequency level required for conducting countercyclical policy, can best be achieved by following a two-step approach – first by fostering coordination between major advanced economies and SMICs, that is, the countries with the largest stakes in the world economy and the strongest scope for influencing each other, through both spillovers and spillbacks, and then, in a second stage, by strengthening coordination with smaller economies.^10^ To a significant extent, this process may be facilitated by the fact that SMICs, through their membership of major international financial institutions, the participation of their central banks in the bi-monthly meetings of the Bank for International Settlements (BIS) and their prominent role in the Group of Twenty (G20), are well positioned to influence global governance issues, and possibly make coordination arrangements more sustainable.^11^

The remainder of this paper proceeds as follows. Sections 2 and 3 set the stage by reviewing the transmission channels associated with, and the empirical evidence on, global financial shocks, in terms of both financial spillovers and spillbacks. Limitations of the literature are also identified. Section 4 evaluates the potential gains from international macroprudential coordination in responding to these shocks, dwelling on both recent analytical contributions and quantitative studies based on multi-country dynamic general equilibrium models with financial market frictions.^12^ We also discuss whether international coordination of macroprudential policies should simultaneously involve some degree of monetary policy coordination, given that these instruments may be complementary in promoting jointly macroeconomic stability and financial stability. Sections 5 and 6 consider preconditions for and practical ways to promote cross-border macroprudential policy coordination. Basel III’s Principle of reciprocity and ways to improve it are examined first. A broader discussion of the role of multilateral institutions is then conducted. The final section brings together the key policy lessons that can be drawn from the analysis.

## International financial spillovers: transmission channels

Understanding the nature and magnitude of financial spillovers and how they are transmitted across borders has been the subject of a large body of literature in recent years. From the perspective of this paper, such understanding is an essential step for assessing the potential benefits of international macroprudential policy coordination. This section begins by defining the nature of financial spillovers. It then describes the various channels, direct and indirect, through which they are propagated internationally. Given the focus of our analysis, particular emphasis is put on the role of cross-border, bank-related capital flows and arbitrage incentives created by domestically focused financial regulation.

### Nature of financial spillovers

Cross-border financial spillovers are commonly defined as occurrences where fluctuations in the price of an asset in one country (or region) trigger changes in the prices of the same asset or other assets in another country (or region).^13^ These fluctuations can reflect both desirable effects (resulting, for instance, from the incorporation of news into forward-looking asset prices) and less desirable ones (such as the transmission of excess volatility due to market distortions and financial frictions). This definition implies that the qualitative nature, and quantitative impact, of cross-border financial spillovers depend on several dimensions: *a*) the type of shocks that generate fluctuations in asset prices in the source country; *b*) the channels, real and financial, through which shocks are transmitted internationally; *c*) the amplification or mitigation mechanisms operating in source and recipient countries; *d*) the nature of the macroeconomic and macroprudential policy regime in source and recipient countries; and *e*) the scope for policymakers in recipient countries to respond in a timely fashion to shocks initiated in source countries.

### Transmission channels

The cross-border transmission of financial shocks (triggered, for instance, by a temporary change in short-term policy interest rates in advanced economies or a sudden shift in market risk perceptions) may occur through a number of conventional channels. In addition, and particularly important from the perspective of this paper, recent studies have emphasized the role of cross-border banking (both as a direct conduit for the propagation of financial shocks, and an amplifying mechanism for these shocks) as well as leakages associated with differences in financial regulatory regimes across countries.

#### Conventional channels

The conventional channels through which financial spillovers are typically deemed to occur involve direct and indirect changes in financial prices, cross-border balance sheet exposures, information or confidence effects (including fundamentals-driven changes in expectations), trade linkages, and policy spillovers.

Spillovers via *asset prices and portfolio effects* represent the standard channel through which financial shocks are transmitted across borders. When financial markets are globally integrated, changes to prices on any asset market usually translate quickly into changes in asset prices and valuations in other economies, through arbitrage and risk premia effects. For instance, when monetary policy is eased in a core country, it tends to lower longer-term yields and to raise other asset prices in that country. Through portfolio balance effects among financially interconnected economies, this may lead to capital flows to, and lower yields and higher asset prices in, other countries. In turn, this may ease financial conditions in these countries. Thus, this channel may operate solely through portfolio reallocation by investors operating in several markets across countries, that is, cross-border financial flows; it does not necessarily depend on the existence of shared fundamentals between source and recipient economies—a phenomenon referred to generally as contagion.^14^,^15^

Spillovers via *cross-border balance sheet exposures* occur through the impact of changes in asset prices on balance sheets. If collateral values depend on the behavior of asset prices (as is the case with house prices) and if changes in collateral values determine access to credit (because real estate is used to secure loans) these effects can be large and affect both consumption and investment. In addition, the wealth effects associated with changes in asset prices can affect household consumption. For banks, a balance sheet weakening can also affect lending capacity.

Spillovers through *trade linkages* occur even if trade flows are considerably less volatile than financial flows – thereby preventing rapid transmission and amplification of shocks through large changes or reversals. In general, trade linkages operate through an income effect and a competitiveness effect (relative price changes), which can work in opposite directions (see, for instance, Ammer et al. (2016)). To the extent that financial shocks affect income (as noted earlier), they may also be amplified through changes in trade flows. Thus, a high degree of trade openness may facilitate the propagation of financial shocks across highly integrated economies.

Spillovers through *information or confidence effects* occur when perception or anticipation of changes in economic fundamentals by market participants are driven by policy announcements (or expectations of them) rather than the actual realization of these changes. They are important for explaining contagion effects, in particular in the context of *wake-up call* effects, which happen when new information concerning a country (or region) induces markets to reassess the vulnerability of other countries (or regions).

Finally, *policy spillovers* occur when domestic monetary and fiscal decisions in source countries have the potential to affect foreign financial variables not only indirectly (through the channels outlined earlier) but also directly, if policymakers in recipient countries respond in the same direction. In particular, to the extent that shocks to world interest rates are accommodated by lower domestic rates, they may generate large spillover effects by inducing domestic banks to borrow more, which in turn would increase their capacity to lend. Thus, the magnitude of financial spillovers depends also on the nature of policy responses, which itself depends on the degree of financial interconnectedness.

**Fig. 2:**
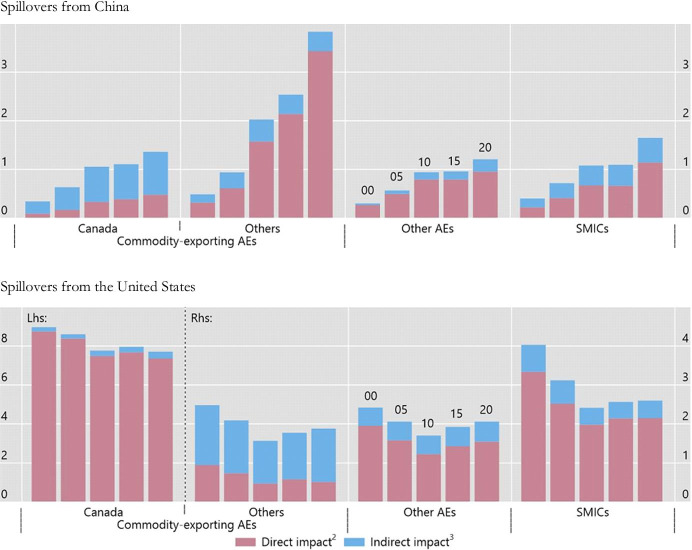
Impact of a 10% Increase in Imports by China and the United States on Total Exports of a Given Economy or Group of Economies (Ratios for 2000, 2005, 2010, 2015 and 2020, in percent). Notes: ^1^SMICs include Brazil, China, India, Indonesia, Mexico, Russia, South Africa and Turkey; however, China is not part of that group in the top panel (only the bottom panel). The United States are not part of other AEs in the bottom panel. ^2^Shares of exports to China/the United States in the respective economies, multiplied by 10%. ^3^Direct effect of the respective economies, multiplied by the corresponding export shares. Sources: IMF, *Direction of Trade Statistics*; BIS calculations

Although by their very nature trade flows tend to be less responsive to global financial shocks than capital flows, could they represent a significant transmission and amplifying channel? In particular, could they account for greater intensity of spillbacks from SMICs to advanced economies and the rest of the world? This is an important issue because (as noted in the introduction) SMICs now account for a significant share of world trade. China, specifically, accounts for a growing share of many countries’ exports, especially in the case of commodity exporters, and the impact of an increase in its spillover effects on them has grown over time (see Fig. 2).^16^ And in contrast to the significant rise in exports destined to China, the share of most countries’ exports to the United States has remained stable or declined somewhat over the past 15 years. Despite this, US demand is still more important than China’s for most countries’ exports. Moreover, trade spillovers can also occur through a third country that imports intermediate inputs used in the production of its own exports. As a result, for many advanced and commodity-exporting SMICs, the indirect impact of a reduction in US imports is large relative to the direct effect. Spillovers from other major advanced economies also remain important for both advanced and systemic middle-income economies. The trade channel appears therefore to account for only a relatively small fraction of the spillback effects associated with global financial shocks.

More generally, the foregoing discussion suggests that financial spillovers and spillbacks are not necessarily bad if they allow new information about changes in economic fundamentals to be reflected accurately in asset prices across different countries. However, they may be undesirable when they contribute to the propagation of shocks across countries – even in the absence of significant economic linkages among them. This is the case, for instance, if portfolio re-balancing considerations induce fund managers in a core country to sell assets in a periphery country, as a result solely of constraints on exposure that they may face. Moreover, in addition to the degree of financial market integration and international portfolio diversification, the nature of policy responses, and the other factors highlighted earlier, the magnitude of financial spillovers may also depend on the cross-border activity of multinational banks and the nature of the regulatory regime in individual countries – two critical dimensions from the perspective of this study.

#### The role of global banks

Between the mid-1990s and the onset of the GFC, cross-border lending and investment activities of banks increased substantially. To a significant extent, this reflected a greater direct provision of loans and financial services by global banks, a greater share of foreign assets in banks’ trading books, and a proliferation of cross-border branches and subsidiaries, which in turn facilitated the provision of loans, investments and financial services. Indeed, as documented by Claessens and van Horen (2014), McCauley et al. (2015), and Claessens (2017), there are now large and growing networks of foreign branches and subsidiaries centered on global parent banks.^17^

**Fig. 3:**
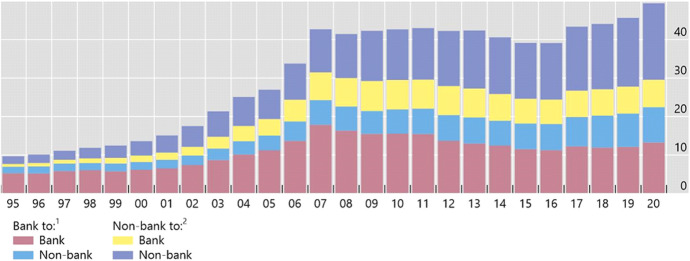
**All Countries: Total Cross-Border Liabilities by Counterparty, 1995–2020** (Trillions of U.S. dollars). Notes: ^1^Cross-border claims in the form of loans and deposits of reporting countries’ banks on all countries. ^2^International debt securities; recipient (lender) sector is assumed to be the non-bank sector. Sources: BIS debt securities and locational banking statistics; BIS calculations

**Fig. 4:**
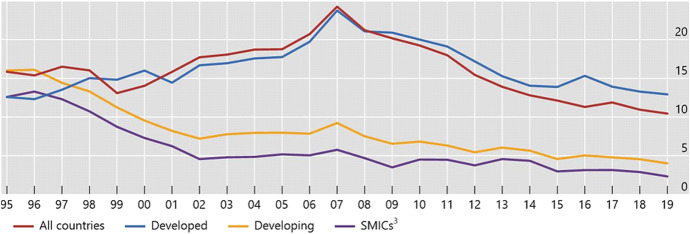
**Various Country Groups: Cross-Border Bank-to-Bank Liabilities, 1995–2019**^1^. (As a percentage of private credit)^2^. Notes: ^1^Cross-border claims in the form of loans and deposits of all reporting banks in the different regions shown. ^2^Domestic credit to the private sector is defined as financial resources provided to the private sector by financial corporations (monetary authorities, deposit money banks and other financial corporations, such as leasing companies, money lenders, and insurance corporations, among others). This definition corresponds to the World Bank’s. ^3^Brazil, China, India, Indonesia, Mexico, Russia, South Africa and Turkey. Sources: World Bank, *World Development Indicators*; BIS locational banking statistics; BIS calculations

Figure 3 shows the classification of cross-border debt liabilities by type of counterparty (banks to banks, banks to non-banks, non-banks to banks, and non-banks to non-banks). It shows that cross-border liabilities where both creditor and debtor are banks are the largest of the four possible categories, and increased rapidly in the run-up to the GFC. Moreover, cross-border bank-to-bank funding (liabilities) can be decomposed into two distinctive forms: *a*) arms-length (interbank) funding that takes place between unrelated banks; and *b*) related (intragroup) funding that takes place in an internal capital market between global parent banks and their foreign affiliates (Reinhardt and Riddiough (2014)). Figure 4 shows that cross-border bank-to-bank liabilities also played a significant role in the expansion of domestic lending, particularly in advanced economies. At their peak, in 2007, these flows accounted for more than 25% of total private credit in recipient countries.

A number of studies have documented that cross-border bank capital flows, increasingly channeled through a small group of large, global banks, have played a significant role in the international transmission of global financial shocks, including during the GFC (see, for instance, Ahrend and Goujard (2015), Buch and Goldberg (2015, 2017), and Claessens (2017)), with sizable macroeconomic and financial effects in countries where these banks operate (see Aldasoro et al. (2020)). These studies have also highlighted two main channels through which the transmission of financial risks can occur. First, if domestic lending standards are relaxed at the same time that cross-border lending is increasing (a common occurrence when banks are awash with liquidity), it can weaken the balance sheets of borrowers in recipient countries and heighten systemic risks there. Second, a global financial institution experiencing difficulties in one of the countries where it operates may fuel financial instability in the other jurisdictions where it is present. In a sense, cross-border banking may create a *credit spillover channel*, which may increase financial vulnerability.^18^

#### Macroprudential policy leakages

It has also become increasingly clear that, in a financially integrated world, macroprudential measures taken in some countries can spill over to other countries through cross-border lending and capital flows—a phenomenon that has been referred to as *policy leakages* (Aiyar et al. (2014a) and Bengui and Bianchi (2018)). For instance, following a tightening of macroprudential restrictions (such as a lower loan-to-value ratio) at home, domestic banks with a regional or global presence may respond by increasing their lending abroad. If increased lending contributes to a credit boom or asset price pressures in the recipient economy or economies, a counterbalancing macroprudential response by regulators there may also be called for to mitigate heightened financial risks – especially if they are in the expansionary phase of their financial cycles.

The credit spillover channel through which cross-border arbitrage by foreign banks may occur can operate not only through direct lending to foreign-country borrowers (firms or households) but also through local lending to foreign branches, as well as a “rebooking” of loans, whereby loans are originated by subsidiaries, but then booked on the balance sheet of the parent institution. Leakages can be to banking institutions not directly covered by the specific policy instrument (Aiyar et al. (2014a)), to shadow banks (Claessens et al. (2021)), or to activities in other geographic regions (Houston et al. (2012) and McCann and O’Toole (2019)). Regardless of the precise channel through which these leakages occur, the presence of foreign branches of financial institutions that are not subject to host country regulation may undermine domestic macroprudential policies.^19^ Thus, the relationship between macroprudential policies and international capital flows can go in both directions: not only are these policies responsive to capital flows, they may also affect these flows. Moreover, these interactions may generate undesirable international spillovers, thereby creating or exacerbating challenges in terms of both macroeconomic and financial stability.

Yet, this is not an unavoidable outcome. While measures aimed, for instance, at limiting risk-taking in a given country could lead to the relocation of risky financial activities to other countries, thereby making them more vulnerable to global financial shocks, macroprudential regulation may also generate *positive* cross-country spillovers. Indeed, if a country becomes more resilient to global financial shocks owing to macroprudential regulation, other countries may enjoy greater stability through less volatile trade and financial flows with that country. More importantly perhaps, policymakers typically have access to a range of tools to respond to macroprudential policy leakages and associated capital flows. From the perspective of an individual country, one option is for prudential authorities to avail themselves of supervisory power over both branches and subsidiaries of foreign banks, and impose their own uniform oversight (including on minimum capital requirements) on all lenders operating within the country and cross-border lending. However, if such uniform oversight is effective, the consequence may be simply to induce foreign financial institutions to shift their activities to other, less regulated countries – which may ultimately be costly for the world economy. The solution to this dilemma, as advocated by some, is harmonization of regulations across countries. But even with a high degree of coordination in setting regulatory standards – an issue we return to later on – banks facing stricter regulation in their home market may still end up taking greater risks in foreign markets, by weakening, for instance, lending standards. These effects are magnified when strict oversight in the home market is coupled with weak supervision abroad (Raman et al. (2016)).

## Evidence on international financial spillovers

Over the last two decades, the empirical evidence on the importance of financial spillovers and spillbacks has grown significantly. Many of the recent studies have focused on the transmission of financial shocks across equity, foreign exchange, and sovereign bond markets, as well as interest rate and balance sheet effects.^20^ This section begins with a brief overview of these studies. In line with our earlier analytical discussion of the transmission channels of global financial shocks, we devote more attention to the evidence on the credit spillover channel and cross-border bank flows, as well as regulatory leakages. Challenges in measuring financial spillovers are subsequently discussed.

### Asset price movements and bond spreads

A common approach to measuring financial spillovers is in terms of the impact of domestic asset price movements on asset prices in other economies. Among the notable studies of this type are those of the IMF ( 2016a, 2016b, 2016c).^21^ In IMF ( 2016a) spillovers are estimated using a vector autoregression (VAR) model of daily asset returns incorporating global control variables. The results suggest that over the last 20 years, spillovers of emerging market asset price shocks to equity prices and exchange rates in advanced and (other) emerging market economies have risen substantially, and now explain over a third of the variation in returns in these countries. In the years immediately following the GFC, average equity market spillovers from emerging market economies (essentially through the portfolio channel discussed earlier) increased by about 28%. These effects also differed significantly across countries; while financial market spillovers from financially open countries, such as Brazil, grew at an even faster pace, spillovers from other economies with financial markets that are less integrated internationally, such as China and India, remained quantitatively limited. For these countries, spillovers from major advanced economies are still stronger than the spillbacks that they may exert on the rest of the world.^22^

**Fig. 5:**
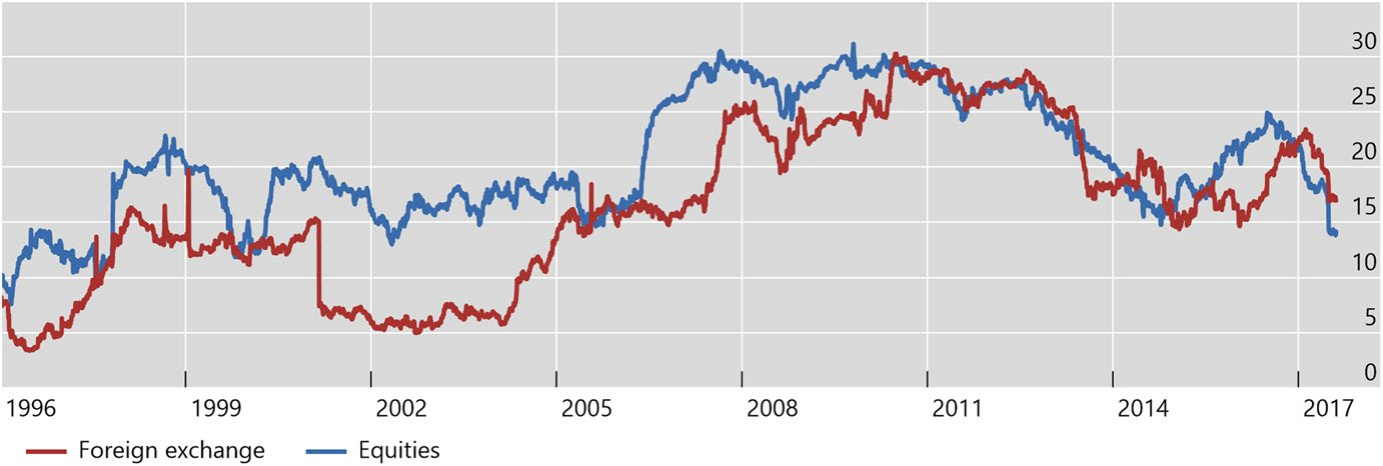
**Spillover from SMICs to an Average Advanced Economy, 1996–2017. Exchange Rate and Equity Returns**^**1**^ (In percentage points). ^1^SMICs: Brazil, China, India, Indonesia, Mexico, Russia, South Africa and Turkey; Advanced economies: Australia, Canada, Denmark, the euro area, Hong Kong SAR, Israel, Japan, Norway, Singapore, Sweden, Switzerland, the United Kingdom and the United States. Financial market spillovers are defined as the fraction of the 12-day-ahead forecast error variance of a country’s local currency nominal equity return that can be accounted for by innovations in another country’s equity return. A similar definition holds for foreign exchange returns. Sources: Bloomberg; BIS calculations

Using the same methodology as the IMF, Fig. 5 shows spillovers from SMICs (as defined earlier) to an average advanced economy, with respect to exchange rate and equity returns. It suggests that, despite significant fluctuations over time, these spillovers appear to have increased on average since 2004 compared to the earlier period of 1996–2003 – especially for currency returns.

The results of studies based on sovereign bond spreads are also consistent with a growing importance of portfolio flows as a conduit for financial spillovers. In particular, there is robust evidence of contagion – as captured by co-movements in measures of bond return risk premia that are unrelated to economic fundamentals – which reflect spillovers driven by exogenous global shifts in risk preferences.

### Interest rate and balance sheet effects

**Fig. 6:**
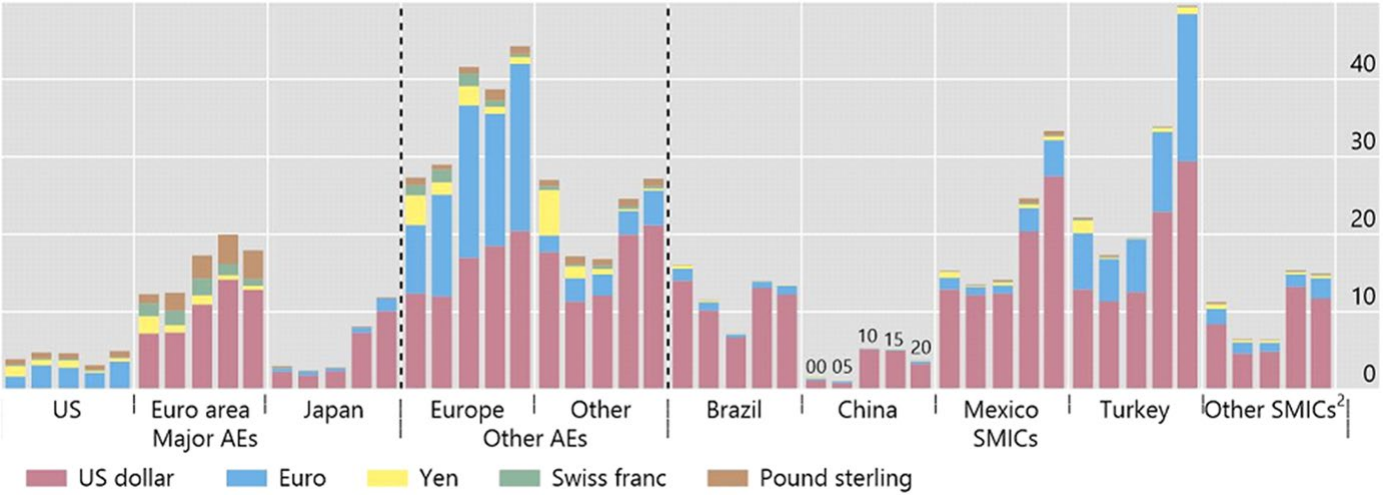
**Selected Countries and Regions: Ratio of Total Foreign Currency Debt to GDP.** (2000, 2005, 2010, 2015 and 2020, in percent). Notes: ^1^Total foreign currency debt of non-bank residents of the respective economies. Simple average across regions. End-of-year ratios. ^2^India, Indonesia, Russia and South Africa. Source: Bank for International Settlements

Fluctuations in interest rates in major advanced economies tend to affect other countries through changes in the cost of external borrowing. For major middle-income countries, whose corporations and banks borrow heavily abroad mostly in US dollars and with little hedging – unlike other advanced economies – changes in US interest rates are a critical channel for financial spillovers. Indeed, Fig. 6 shows that SMICs have relatively high ratios of foreign currency debt to GDP. Financial spillovers may therefore amplify domestic leverage and generate large effects when borrowers face financial distress.

At the same time, the accumulation of a large stock of foreign currency-denominated debt in SMICs has heightened the potential for spillbacks to advanced economies. As noted in the introduction, low US interest rates and a depreciating US dollar boosted credit, asset prices and growth in SMICs in the aftermath of the GFC. A tightening in global financial conditions induced by prospects of higher US interest rates (as is the case at the time of this writing) could trigger a reversal of easy liquidity conditions for SMICs. Spillovers to advanced economies from SMIC holdings of specific advanced-economy assets, such as sovereign bonds, have increased (Bank for International Settlements (2016)). By contrast, spillovers to advanced economies through wealth effects from direct ownership of SMIC assets are generally small, in line with the share of SMIC assets in advanced economy portfolios.

Studies based on transmission through policy rates include Hofmann and Takáts (2015), who used quarterly panel data regressions over the period 2000–14.^23^ In all their specifications, they explicitly controlled for the impact of domestic and global macroeconomic factors, as well as global financial factors. They found economically and statistically significant spillovers from the United States to a range of countries. These spillovers are present not only in short- and long-term interest rates but also in policy rates.^24^

### Cross-border bank flows and the credit spillover channel

Evidence on the determinants and effects of cross-border bank flows and the credit spillover channel is provided in a number of recent contributions. Studies by Cetorelli and Goldberg (2012), Bruno and Shin (2015), Tonzer (2015), Cerutti et al. (2017a) and Correa et al. (2018) focused on aggregate banking flows, whereas Reinhardt and Riddiough (2014) zeroed in on disaggregated (interbank and intragroup) flows. By and large, these studies have shown that cross-border bank capital flows are highly sensitive to changes in interest rates in advanced economies and changes in global risk perceptions, and that these changes tend to operate quickly – with potential consequences for financial stability in destination countries. Tonzer (2015), for instance, found that countries that are linked to more stable banking systems abroad through foreign borrowing or lending positions in interbank markets are significantly affected by positive spillover effects. Thus, in times of financial volatility, linkages in the banking system can contribute to the propagation of shocks. For their part, Correa et al. (2018) provided empirical support for the existence of an international portfolio rebalancing channel, whereby tighter monetary policy in source countries leads to a decrease in collateral values and the net worth of domestic borrowers, which prompts banks to substitute away from domestic credit and toward foreign credit. In a study focusing on disaggregated flows, Reinhardt and Riddiough (2014) found that intragroup funding appears to be unrelated to global or local cyclical factors, whereas interbank funding appears to respond procyclically. Thus, the *composition* of bank funding may also matter for the cross-border transmission of financial shocks.

### Regulatory leakages and capital flows

Studies focusing on how regulatory leakages affect cross-border capital flows, which therefore act as a conduit to financial spillovers, include Houston et al. (2012), Bremus and Fratzscher et al. (2014), Aiyar et al. (2014a, 2014b), Karolyi and Taboada (2015), Reinhardt and Sowerbutts (2016), Avdjiev et al. (2017), Beirne and Friedrich (2017), Cerutti et al. (2017a), Forbes et al. (2017), Kang et al. (2017), and Takáts and Temesvary (2017).^25^ Houston et al. used data aggregated at the country level and survey data on global regulations to argue that cross-border banking flows move to circumvent regulations. In the same vein, Bremus and Fratzscher found that inflows and outflows of international capital through banks around the time of the GFC responded to the stance of regulation and supervision. Using a broader sample covering the period 2000–14, Avdjiev et al. found that changes in macroprudential policy – in the form of loan-to-value limits and local-currency reserve requirements – have a significant impact on cross-border bank lending. Similar results are obtained by Takáts and Temesvary. Using also a large sample of countries and a broader variety of empirical techniques, Kang et al. found that while sectoral and liquidity based macroprudential policy measures tend to generate large cross-border bank credit spillovers, that is not the case for capital-based measures. Finally, using a sample of countries twice as large as Kang et al., Beirne and Friedrich (2017) examined the impact of eight different macroprudential policy measures on cross-border bank flows over the period 1999–2012. They found that the magnitude of cross-border spillovers associated with these measures were a function of banking sector conditions, both at home and abroad. In particular, in countries that are financially healthy (as measured by a high return on assets in the banking system), the magnitude of spillovers from the one implementing the policy measures was weaker.

Empirical evidence on cross-border spillovers associated with prudential measures has also been provided in the context of individual countries, both within the country where a macroprudential policy instrument is applied to domestic banks but not to foreign institutions competing in the same markets, and through reallocation of activity between domestic and international locations. Two important studies in that regard are those of Aiyar et al. (2014a, 2014b) for the United Kingdom, which focused on changes in capital requirements imposed by the national financial regulator (the Financial Services Authority, or FSA) on banks in the country. In the period under study, subsidiaries of foreign banks located in the United Kingdom were supervised by the FSA, whereas foreign bank branches in the country remained under the oversight of the authorities in their home country. Aiyar et al. found that when the FSA raised minimum capital requirements, there were substantial leakages, in the sense that lending shifted, from local banks and subsidiaries, to foreign-supervised branches located in the United Kingdom. They estimated the UK-wide leakages (offset) on domestic credit growth, owing only to bank branches, to be of the order of 30%. In the same vein, Reinhardt and Sowerbutts (2016) found in a broader study that cross-border leakages appear strong as well for capital requirements, especially in countries where affiliates are established as branches, but weaker for loan restriction instruments, such as loan-to-value and debt-to-income ratios. Focusing on the euro area, Franch et al. (2019) found that foreign affiliates increase lending following a tightening of sector-specific capital buffers in the countries where their parent banks are located, and that bank size and liquidity play a significant role in determining the magnitude of cross-border spillovers.

Finally, some indirect evidence on cross-border spillovers is also available. Karolyi and Taboada (2015), for instance, studied how differences in bank regulation influence bank acquisition flows across countries and share price reactions to cross-border deal announcements. Using a large sample of domestic and majority cross-border deals announced between 1995 and 2012, they found evidence of a form of regulatory arbitrage, whereby acquisition flows involve acquirers from countries with stronger regulations than their targets. They also found that target and aggregate abnormal returns around deal announcements are positive and larger when acquirers come from more restrictive bank regulatory environments.

### Challenges in measuring financial spillovers

The recent empirical literature discussed earlier provides convincing evidence of the increased importance of international financial market spillovers through bank and nonbank capital flows – especially those associated with portfolio reallocation or cross-border regulatory arbitrage. However, there are several dimensions in which the empirical literature can be improved. First, there is a need to examine domestic effects and international spillovers using more detailed micro-banking data, and to develop more precise measures of prudential regulation than were initially available to researchers studying cross-border spillovers. More recent databases on prudential instruments compiled by Cerutti et al. (2017b), Akinci and Olmstead-Rumsey (2018), and Alam et al. (2019), for instance, which put greater emphasis on the *intensity*, and not simply the *direction*, of macroprudential policy instrument use, are proving useful in that regard.

Second, for the most part existing econometric studies analyze the cross-border spillover effects of different shocks separately, which is useful to focus on their specific transmission mechanisms. However, in practice these shocks rarely occur in isolation, implying that there can be important interaction effects – which in turn may determine the magnitude and direction of spillover effects. For instance, shifts in global risk aversion may affect not only asset prices across countries but also world commodity prices, which may magnify interactions between trade and financial flows. An appropriate methodology is thus needed to better account for these interactions and assess their implications for cross-border spillovers and spillbacks, conditional on the nature of the macroprudential regime and the nature of global shocks.

Third, a better distinction between common shocks versus spillovers of country-specific shocks is needed. To differentiate co-movement due to spillovers from co-movement due to common and/or correlated shocks and permanent country-pair differences, spillovers are often identified with the transmission of a country-specific shock (or policy action) to other countries. To do so requires conditioning on common shocks. The challenge, however, is to separate common shocks from propagation of country-specific shocks through different channels. There may be direct spillovers (for instance, from the United States to Brazil) as well as indirect spillovers (for instance, from the United States to China, and subsequently from China to Brazil). To allow for the possibility of second-round spillovers, it is thus necessary to analyze multiple countries, or groups of countries, simultaneously. In the present setting, a basic framework would involve considering three groups -- advanced economies, SMICs (given their growing role in the world economy, as noted earlier), and the rest of the world as an aggregate.^26^ One issue to be tested in that context is whether financial market spillovers between the first two groups of countries are, or have become, quantitatively stronger than real spillovers, and whether financial shocks have a disproportionate effect on cross-border real and financial spillovers during crisis periods.

## International macroprudential policy coordination: rationale and potential gains

The scope for international macroprudential policy coordination to mitigate the adverse effects of cross-border financial spillovers and raise global welfare has been the subject of much interest in recent years. This section begins with a brief review of the link between systemic financial risks and the rationale for macroprudential regulation. The fundamental case for cross-border macroprudential policy coordination is discussed next. Quantitative evidence on the gains from cross-border macroprudential policy coordination is then examined. The section concludes with a discussion of whether monetary and macroprudential policies should be simultaneously coordinated across borders to some degree to be effective.

### Systemic risks and the rationale for macroprudential regulation

The goal of macroprudential policy is commonly described as promoting financial stability by mitigating systemic risks to the financial system.^27^ This contrasts with microprudential supervision, which focuses on the financial health of individual financial institutions. Systemic risks fall into four broad categories: excessive credit growth (often associated with procyclical risk-taking by financial institutions) and associated asset price inflation; excessive leveraging or deleveraging; systemic liquidity risks; and large and volatile capital flows. These risk categories often occur in combination with each other, and to varying degrees. In particular, SMICs have time and again been confronted with episodes of sudden floods in capital flows, rapid credit growth, asset price pressures, and excessive leveraging – followed by sudden stops in capital movements, which would throw the previous process into reverse (Agénor (2020, chapter 1) and Agénor et al. (2014, 2018)).

There is broad consensus that, from an operational standpoint, an aggregate that may serve as a proxy for financial stability is credit growth or changes in the credit-to-GDP ratio. On that basis, credit can be viewed as a “summary” indicator or intermediate target, which can be used to calibrate the effect of macroprudential instruments and design policies to dampen destabilizing swings in the credit cycle.^28^

However, despite significant progress in recent years, no consensus has yet emerged on the transmission mechanism and effectiveness of macroprudential policies, their complementarity with microprudential policies, and the degree to which they should be coordinated with monetary policy – given that the regulatory regime may alter the monetary transmission mechanism and that changes in macroprudential instruments, through their impact on the cost and the availability of credit, can affect activity and prices.^29^ Moreover, as documented in the previous section, cross-border activities of financial institutions may exacerbate challenges to macroprudential policies, with possibly unwelcome spillover effects weakening their domestic policy impact.

It is also well documented that macroprudential policy (just like many other policies) can be subject to a collective action problem, which arises from the existence of uncertainty over the benefits of macroprudential action, lobbying, and political pressure. This translates into the well-known challenge of “taking the punch bowl away just as the party gets going,” which makes the containment of financial excesses politically difficult at a national level. Thus, more often than not, the collective action problem results in too little, rather than too much, macroprudential policy response, relative to the level that would be necessary to promote financial stability or maximize national welfare. Indeed, as noted by Viñals and Nier (2014), while the benefit of macroprudential policy decisions typically accrue over time and can be difficult to measure with certainty, the cost of such decisions is often felt immediately by both borrowers and lenders. This makes it hard for financial regulators to demonstrate the will to intervene. The resulting bias in favor of inaction, or insufficiently timely and forceful action, is often compounded by lobbying and political pressure, as well as the need for domestic coordination between policymakers (say, the financial regulator and the central bank) and a lack of clarity about who is responsible for containing systemic risks. As discussed next, these problems tend to be magnified in a financially integrated world.

### Fundamental case for cross-border macroprudential policy coordination

A fundamental rationale for policy coordination is the existence of externalities.^30^ The literature has identified three types of externalities that might require coordination: those based on incomplete or asymmetric information, those due to asymmetries in incentives, and those due to spillovers (across agents or jurisdictions) associated with specific shocks or policies. These externalities could be either positive or negative, which implies that coordination could either prevent welfare losses or achieve welfare gains. The scope for coordination exists if there are cooperative strategies that could result in a Pareto-improving outcome.

For macroprudential policy in particular, the case for international coordination rests on the existence of cross-border financial sector externalities, related to interconnectedness of financial institutions and markets. As documented earlier, when regulatory leakages occur, the effectiveness of macroprudential policy measures applied solely to domestic financial institutions may be undermined by cross-border capital flows. Moreover, capital inflows induced by changes in financial regulation in a source country may lead to excessive credit growth and asset price pressures in recipient countries, which may only be partially mitigated by regulatory and macroeconomic policy responses in these countries. Conversely, and as noted earlier, effective domestic macroprudential policy that helps to contain systemic risks in one country may help to promote financial stability elsewhere, creating positive externalities. Indeed, lowering the probability of a financial crisis in one country through timely macroprudential policies may reduce the scope for negative trade and financial spillovers at the regional or international level. Thus, coordination is desirable when it enables countries to improve their policy trade-offs (Engel (2016)).

#### The financial trilemma

A broader case for macroprudential policy coordination among small open economies, regardless of the exchange rate regime, rests on what Claessens et al. (2010, Chapter 2), Schoenmaker (2011), and Obstfeld (2015), referred to as the *financial trilemma* – the fact that financial integration with global markets (with unrestricted cross-border financial flows), national control over financial supervision and regulation, and financial stability, are not all mutually compatible. That is, under a financial trilemma, a country can attain any pair of these goals: financial stability and international financial integration, financial stability and independently pursued financial policy making, or international integration and autonomous financial regulatory policies.^31^ However, all three objectives cannot be achieved simultaneously.

The financial trilemma implies that, should countries choose to focus on domestic financial stability and to pursue an independent financial policy – assuming that macroprudential policies are effective in dealing with the various dimensions of financial stability issues – the goal of full integration with international financial markets cannot be achieved. National banking authorities may face significant pressures to insulate their financial systems from international competition. Such a strategy may result in financial protectionism (VanHoose (2016)). It may also imply a *race to the bottom*, involving unilateral capital controls (as discussed by Blanchard (2017)) or *regulatory wars* (as pointed out by Pereira da Silva and Chui (2017)), both of which would be detrimental to world welfare. By contrast, coordination among national regulators, which induces them to internalize the cross-border effects of their policies, may help to avoid these risks.^32^ Thus, as long as obstacles to cooperative agreements can be mitigated in practice (an issue we return to later on), coordination may help to address the trade-offs highlighted by the financial trilemma.

#### Financial spillovers and international collective action problems

As noted earlier, macroprudential policy is subject to collective action problems, which often translate into insufficiently forceful and timely policy responses. In a financially integrated global economy, characterized by a high degree of cross-border interconnectedness of financial institutions and markets, these problems tend to be magnified. This is so, for instance, as a result of the *first-mover disadvantage problem*, which is caused by an inability of national regulators to internalize the cross-border externalities that a successful national macroprudential policy entails. In a world of integrated financial markets, a reduction of financial risks in an individual country contributes to financial stability in other countries (positive externality), whereas an inadequate response by a national regulator to home-country financial risks may increase the likelihood that financial instability may spread to other countries (negative externality). For instance, in a period of rapid credit growth and asset price pressures, a national regulator which chooses unilaterally to tighten its macroprudential policy (through, say, an across-the-board increase in capital requirements) would internalize only *some* of the financial stability benefits of this tightening while bearing all the potential costs in terms of reduced competitiveness of its national financial institutions.^33^ Thus, no country will be willing to be the first to tighten its policies unless it believes that other countries are willing to do the same. When financial risks can be transmitted rapidly across national borders, and macroprudential measures – when applied solely to domestic financial institutions – can be promptly undermined by large capital movements across countries, there may be “too little” macroprudential policy response rather than “too much.” This may in turn reinforce biases in favor of inaction at the national level (Gaspar and Schinasi (2010) and Viñals and Nier (2014)).

Another type of collective action problem in an international context is the fact that advanced economies may claim that their mandate is narrowly defined to promote price stability and sustainable growth at home, which requires taking account of the external impact of their policies only insofar as they feed back onto their own economies. That is, only spillbacks, not spillovers per se, need to be internalized. Moreover, even when each country’s macroprudential policy is optimal at the national level, the overall combination of macroprudential policies may be suboptimal when financial cycles are not synchronized across countries or, as noted earlier, when global financial intermediaries can evade policy actions taken by national authorities through cross-border regulatory arbitrage.

What the foregoing discussion suggests is that, to reap the benefits of financial integration and ensure global financial stability, it is essential to implement measures designed to mitigate collective action problems at both the national and international levels, make regulatory arbitrage across borders more difficult (including by ensuring a high degree of international reciprocity of national macroprudential legislations, as discussed next), and establish cooperative mechanisms that can help to mitigate the financial risks that may be building up in systemic countries – including through international coordination of countercyclical macroprudential policies.^34^ But before we can discuss these institutional mechanisms, we need a broader assessment of the gains from, and obstacles to, macroprudential policy coordination.

### Gains from, and obstacles to, macroprudential policy coordination

To a large extent, the discussion of the gains (or lack thereof) associated with international macroprudential regulation has been based on comparing outcomes under a Nash equilibrium, in which countries act independently, and a cooperative solution, in which they act jointly. More formally, under uncoordinated policymaking, each country’s regulatory authority independently sets its policy instrument so as to minimize its own policy loss or maximize its own welfare, taking the choice of instrument of all other countries as given. The resulting policy outcomes typically fail to fully account for cross-border spillovers – that is, the real and financial externalities generated by domestic shocks, or national policy responses to these shocks, as discussed earlier. In contrast, if the regulatory authorities coordinate their choices by jointly determining their instruments with a view to minimizing a weighted sum of their policy loss functions, or maximizing a weighted sum of their national welfare functions, these spillovers would be internalized. As a consequence, and depending on the nature of the cross-border externality, coordination may enable all policymakers to attain lower policy losses or higher social welfare.

In what follows, we begin with a brief review of the analytical literature on cross-border macroprudential policy coordination. We then discuss the recent, model-based evidence on the gains associated with such coordination. We conclude by offering some suggestions for further research on measuring these gains.

#### Analytical contributions

The analytical literature on cross-border macroprudential policy coordination includes two somewhat disconnected but complementary sets of contributions – the first based on partial equilibrium models of international banking and the second (more recent) on multi-country general equilibrium macroeconomic models with financial frictions.

##### Partial equilibrium models of international banking

The banking and finance literature on international prudential policy coordination includes Acharya (2003), Dell’Ariccia and Marquez (2006), and Kara (2016).^35^ DellAriccia and Marquez studied the incentives of national regulators to form a regulatory union in a two-country world, where a single bank from each country competes for loans in both markets in a Bertrand differentiated-products setting. Both regulators focus on the profitability of national institutions, but there is an exogenously specified asymmetry between them in terms of their preferences. The key result of the study is to show that under independent policymaking the outcome, in terms of prudential standards, could well be a *race to the bottom*. By contrast, a coordinated structure with higher prudential standards is more likely to emerge if: *a*) the impact upon profitability of prudential supervision is similar across countries; *b*) the weights assigned by supervisors to financial stability and banking sector competitiveness are similar; and *c*) the weight assigned to financial stability by supervisors is larger than that assigned to profitability and competitiveness.

Acharya (2003) focuses instead on practical issues that may impede cross-border regulatory coordination efforts. His key argument is that convergence in international capital adequacy standards cannot be effective unless it is accompanied by convergence in other aspects of financial regulation, such as bank closure policies. Thus, coordination in setting regulatory standards does not necessarily eliminate regulatory arbitrage – an important result, in light of the recent efforts to promote international cooperation (discussed later on) by the Basel Committee.

Both of the studies referred to above focused on the benefits of international coordination in financial regulation under externalities that operate through integrated loan or deposit markets in stable times. In contrast, Kara (2016) focused on pecuniary externalities between national financial markets that operate through asset markets and asset prices during times of distress.^36^ In his model, banks invest in a single country and are therefore regulated only by their home supervisor – although they interact with each other in global asset markets. Systemic risk in international financial markets arises as banks experience correlated liquidity shocks, and financial amplification effects are triggered by fire sales. Regulators act simultaneously and choose the regulatory standard – a minimum capital ratio requirement, which is macroprudential in nature because it is motivated by systemic externalities – for banks in their own country. His key result is that regulation levels in the two countries are *strategic substitutes*: if one regulator tightens the standards in its jurisdiction, the other regulator optimally loosens its own standards. This follows from the fact that macroprudential regulation in an international context – or, more accurately, the global financial stability that it helps to promote – is fundamentally a public good. Moreover, Kara showed that the capital adequacy ratio in the non-cooperative equilibrium is inefficiently low compared to the ratio that a central regulator would choose. The key reason is that although national regulators internalize the positive effect of tighter capital requirements on asset prices, they have an incentive to free ride on regulations in the other country. Therefore, in a symmetric world, and if a binding commitment mechanism can be implemented, both countries can improve their own welfare by delegating regulatory oversight to a common regulator. However, as discussed further later on, the incentive to renege on cooperative agreements remains a key concern in practice.

Another branch of the literature considers the regulation of a multinational bank that operates across countries. Dalen and Olsen (2003) and Holthausen and Rønde (2004), for instance, focused on the tension between home and host country regulators of a global bank where informational asymmetries are the driving force of regulatory competition. In particular, Holthausen and Rønde examined issues related to information exchange between supervisory authorities, and concluded that centralization of the supervisory process within a supranational authority can yield welfare-improving bank-closure policies.

The banking and finance literature on international macroprudential policy coordination sheds useful light on a number of issues – including the structure of banking markets across countries and the objectives of financial regulators. However, the partial equilibrium nature of these models also means that they are not well suited for a full assessment of the policy or welfare gains associated with international macroprudential coordination – whether in its structural or countercyclical dimensions. To that end, general equilibrium macroeconomic models, which capture the various channels through macroprudential policies operate, are better suited.

##### Macroeconomic models

Recent analytical contributions on the international coordination of macroprudential policies from a macroeconomic perspective include Korinek (2014), Bengui (2014), and Jeanne (2014). Korinek showed that international cooperation is not warranted if small countries can use prudential capital controls to respond to domestic externalities. However, as noted earlier unilateral capital controls could trigger a *race to the bottom*, which would be detrimental to world welfare. Bengui studied the scope for international coordination in a model with public liquidity provision. He found that the noncooperative equilibrium between national regulators leads (in standard fashion) to an inefficiently low level of regulation, as national regulators do not internalize the benefits of their country’s provision of liquidity to the rest of the world. By contrast, Jeanne analyzed the scope for international coordination in a model where both domestic macroprudential policies and prudential capital controls generate international spillovers through their impact on capital flows. The uncoordinated use of macroprudential policies may lead to a “capital war” that depresses global interest rates. However, international coordination of macroprudential policies is not warranted, unless there is unemployment in some countries, or one part of the world is in a liquidity trap, while the rest of the world accumulates reserves for prudential reasons.

##### Obstacles to policy coordination

The banking-based and macro-based analytical literature reviewed earlier is somewhat mixed, in the sense that it suggests that coordinated macroprudential policies can potentially offer significant gains, even though this is not necessarily the case. Moreover, even if gains do exist, achieving and maintaining coordinated policies across countries in pursuit of these gains may prove difficult in practice.

First, suppose that a cooperative outcome can indeed be achieved, and that regulators have agreed to coordinate; each of them almost invariably has an incentive to cheat. Indeed, once one of the countries’ regulators has set its instrument at the agreed level, the others typically can set their own instrument at a different value and attain an even lower policy loss or higher welfare. As in standard game theory models, this incentive is stronger the smaller the perceived *ex post* cost of reneging on a cooperative agreement.

Second, cooperative solutions may be inefficient in the presence of third-party effects: in a policy game with three or more players, the welfare contribution of a subgroup coalition generally cannot be determined a priori, and it is very possible that policy coordination may worsen welfare (see Rogoff (1985) and Cai and McKibbin (2013)). This is important because, as discussed next, recent empirical contributions have generally been based on two-country models, in which a “core country” (which can be interpreted as an aggregate of major advanced economies) and a “periphery country” (which can be interpreted as the group of SMICs identified earlier) operate. However, while a two-country (or two-region) structure may be appropriate to generate analytical insights, as well as broad estimates of the gains from coordination, it does not account for the fact that in practice these groups are not homogeneous and face coordination issues of their own. Among advanced economies, for instance, these issues are equally important between the United States, Japan, and the euro area – even though these countries have in the past cooperated sporadically, often in the context of emergency macroeconomic policy responses to heightened risks to the world economy. This issue is even more problematic in the case of SMICs, given their historical record (or lack thereof) in that area.

Third, simple theoretical models assume that regulators across countries have the same targets and/or common national preferences, whereas in practice supervisory authorities may place diverging weights on similar goals or seek different objectives (see VanHoose (2016)). Fourth, theoretical models often assume that different countries share the same view of the world; however, in practice they often have fundamentally different perspectives on how the world economy operates. Thus, even well-intentioned regulators may not be able to carry on a coherent discussion of the potential gains from coordination – which involves assessing the costs and benefits of alternative policy choices – and how to achieve them. Indeed, as shown by Frankel and Rockett (1988) in the context of the debate on monetary policy coordination, if models are incorrect international coordination could worsen outcomes (by moving policies in the wrong direction) instead of improving them. Moreover, model perceptions could be endogenous with respect to individual country interests.^37^ These problems are compounded if one thinks of policy coordination in terms of countercyclical responses (as opposed to permanent, or structural, settings of macroprudential policy instruments) because agreement on the origin and nature of shocks (common or idiosyncratic, permanent or transitory, and so on) also matters.

For all these reasons, maintaining a macroprudential policy coordination agreement is likely to be challenging in practice – even when mutual net gains from such coordination are potentially large.^38^ A common response to the first challenge to maintaining coordinated policies across countries is to ensure that appropriate and credible sanctions are in place to eliminate the temptation to renege. To address the fourth challenge, a possible response might be for countries to entrust an assessment of the origin and nature of global shocks, and the need for a coordinated international response, to a group of multilateral institutions – in effect, a group of “honest brokers.” This could help not only to address the issue of model uncertainty and the magnitude of policy gains, but also to alleviate some of the collective action problems discussed earlier – inertia in policymakers’ reaction and the disadvantage of moving first –which combine to prevent a timely response to financial risks. This issue is further discussed later on.

#### Quantifying the gains from cross-border coordination

The early empirical literature on the gains from international monetary policy coordination, largely based on multi-country econometric models, has traditionally found gains to be modest.^39^ This could be related to the fact that in these models international goods market spillovers tend to be very small, because a large part of the adjustment to shocks consists of relative price changes – which themselves tend to be relatively limited, especially with sticky prices and a low degree of trade integration.^40^ Indeed, some studies based on alternative assumptions about real linkages between countries, based on calibrated, two-country simulation models did find potentially large gains from international monetary policy coordination (see, for instance, Liu and Pappa (2008)).

However, the most important reason as to why the early literature found only small gains from international monetary policy coordination may well be the fact that for the most part it did not account for various types of capital flows (bank and nonbank related) and largely abstracted from the financial system and its role in magnifying the response to shocks. Capturing the implications of greater international financial integration (including an increased role of global banks, as documented earlier) for capital flows, as well as financial frictions at home and abroad, could potentially make the welfare benefits from monetary policy coordination in response to the cross-border transmission of real and financial shocks significantly larger than the estimates provided by existing contributions.^41^

In the area of macroprudential policy coordination, where contributions have only recently begun to emerge, these features have figured prominently in model design. In addition, some models also account for the fact that macroprudential regimes affect the monetary transmission mechanism – in line with the closed-economy literature on monetary policy.^42^ As noted earlier, the international interconnectedness of financial markets, the possibility that regulatory leakages may weaken the ability of national policies to mitigate financial risks in a world with global financial institutions, and the fact that frictions in national financial systems can amplify the cross-border effects of domestic shocks, suggest indeed that significant gains from coordination may exist.^43^

Nevertheless, model-based contributions focusing on the gains from international macroprudential policy coordination remain scarce. Instead, recent studies have focused more on measuring the magnitude of cross-border financial spillovers themselves, rather than providing quantitative estimates of the gains from coordination.^44^ Among the few contributions available, based explicitly on a game-theoretic approach, are Chen and Phelan (2017), Agénor et al. (2018), and Agénor and Pereira da Silva (2019a).^45^ The first study focuses on the case where financial frictions relate to the inability of countries to issue equity to each other. In that setting, coordinated macroprudential policies (in the form of borrowing limits) improves welfare. More related to our purpose in this paper, the second and third contributions study the gains from international macroprudential policy coordination in a two-region, core-periphery model with a global bank and financial frictions, with periphery banks borrowing from the core global bank to fund domestic lending. Both studies found that these gains, when unconstrained policies are used, are significant. In addition, gains are not equally distributed across countries; depending on the nature of the shock, gains for the periphery can be larger than those accruing to the core region. This could point to potential political-economy obstacles to the implementation of cooperative policies – an issue we return to later on.

#### Challenges in measuring the gains from coordination

The few studies summarized earlier provide several insights into what may affect, quantitatively, the gains from international macroprudential policy coordination. In particular, they suggest that the welfare gains from coordination are stronger when *a*) models are capable of generating large cross-border financial spillovers (as observed in recent years); *b*) financial frictions and financial amplification mechanisms at the level of individual countries are accounted for, as well as asymmetries in financial market imperfections across countries; and *c*) global regulators, entrusted to implement a cooperative solution, are able to internalize the fact that national regulators (despite being subject to collective action problems) may have a higher preference for financial stability. As a result, they may end up putting a higher weight on that objective in the global policy loss or welfare function. In fact, based on the previous discussion of the early literature on policy coordination, a better account of financial linkages between countries (especially through global financial institutions), assuming that they are associated with externalities that are not efficiently priced through movements in interest rates and real exchange rates, may be essential to generate large cross-border spillovers.

At the same time, given that in practice (as noted earlier) disagreement over models may be a significant impediment to coordination, it is important to establish the robustness of these results and to explore other channels that may affect the gains (or lack thereof) from coordination. In particular, the performance of simple rules should be compared with fully optimal policies – even though the latter are often complex and difficult to implement in practice. The idea that the presence of quantitatively important economic nonlinearities and asymmetries, especially in the financial system, may enhance the benefits from international macroprudential policy coordination also needs to be studied further. And the fact that cross-border leakages through global financial institutions can undermine the effectiveness of national macroprudential policies, and potentially magnify the gains from international coordination, should be explicitly accounted for in multi-country quantitative macroeconomic models. Finally, it may be important to use or develop models with more than two countries, to understand (as discussed earlier) how sub-coalitions can weaken or strengthen global gains from coordination.

Without significant progress in these directions, it remains difficult to make a convincing case for international macroprudential policy coordination and to ensure that countries narrow the differences in their “global model perceptions” (or, more generally, their priors on how the world economy works) should they come together to discuss the potential gains associated with coordination and how to secure them. This is especially important given that, as noted earlier in the context of monetary policy, international coordination could make matters worse, rather than better, if the models used for policy analysis and calibration are wrong. As discussed later on, multilateral institutions could play a significant role in this process.

### Should monetary and macroprudential policies be coordinated across borders?

There is now a large amount of evidence to suggest that monetary policy may affect not only price stability but also financial stability, through various channels – including a risk channel, as discussed by Borio and Zhu (2012) and Adrian and Nellie Liang (2018). Indeed, changes in interest rates affect not only aggregate demand and supply but also financial conditions through intermediation costs, asset prices, borrowing and collateral constraints, banks’ balance sheets and risk-taking behavior, and default risks, as well as capital flows and exchange rates. Conversely, it is also well established that macroprudential policy regimes can affect the monetary transmission mechanism – possibly in substantial ways (Agénor and Pereira da Silva (2014)). These interactions have led to an ongoing debate on whether, at the level of the domestic economy, monetary and macroprudential policies are complements in achieving macroeconomic and financial stability. From the perspective of this paper, the issue is whether the same matter arises at the international level. We consider briefly both of these issues.

#### Monetary policy and financial stability

Fundamental to the issue of complementarity between monetary and macroprudential policies within countries is an understanding of the division of tasks between central banks and regulators. Many observers have argued that macroprudential policy cannot be a substitute for sound monetary policy, and that the priority for monetary policy should remain on price stability. At the same time, macroprudential policy’s primary focus should be on containing systemic financial sector risks. Such clear mandates serve to protect the independence that policymakers need to conduct countercyclical policies and simultaneously achieve or maintain price stability and financial stability. Others, however, have argued that there are circumstances where monetary policy may still need to *lean against the wind* and respond to financial sector distortions – because macroprudential policies alone may not be sufficiently effective in containing systemic risks arising from macroeconomic imbalances, as documented by Borio et al. (2021) and Agénor and Pereira da Silva (2014), for instance – whereas macroprudential policy may be needed to attain macroeconomic stability objectives. The view that macroprudential and monetary policies are complements in achieving price and financial stability, and should therefore be coordinated at the individual country level, has gained greater acceptance in recent years.^46^ It has also been supported by some recent empirical evidence.^47^

#### International coordination of monetary and macroprudential policies

The foregoing discussion suggests that, at the level of a single economy, there are some valid arguments regarding the desirability of coordinating macroprudential and monetary policies – given their characteristics, their interactions, and the requirements of financial stability.^48^ In light of this growing consensus, and given the issue at stake, should there also be coordination of these policies at the international level?

The answer to this question is not straightforward. First, although some studies (including Rey (2015) and Miranda-Agrippino and Rey (2020)) have found that US monetary policy is a key global driver in assets prices, risk premia, and other financial variables, the magnitude of this effect has been questioned in others (see Cerutti et al. (2017c) and Arregui et al. (2018)). But even if cross-border spillovers associated with changes in US interest rates are large, it does not follow that monetary and macroprudential policies should also be coordinated across countries; fluctuations in financial variables do not necessarily heighten financial risks – in line with our previous discussion of financial spillovers, the strength of the recipient country’s prudential regime, and the pervasiveness of domestic financial frictions, matter also.

Second, in practice the requirement to coordinate macroprudential and monetary policies presents a greater challenge at the international level. Monetary policy coordination across borders is more difficult because it is often less rules-based and mechanistic than *structural* macroprudential regulation – except in challenging times.^49^ In addition, a policy regime that involves *countercyclical* macroprudential and monetary responses may require timely judgment and decisions, often with partial or incomplete information, compared to a regime involving only systematic responses. The scope for disagreement about the origin and nature of shocks, and how to respond to them, may therefore make it more difficult (as noted earlier) to implement coordinated policies.

In sum, monetary policy can have deleterious effects on financial stability, which in turn may need to be contained by appropriate macroprudential action. This applies both at the national level, when, for instance, accommodative monetary policy contributes to excessive increases in domestic asset prices and credit growth, and at the international level, when changes in the monetary stance of a group of countries can cause spillovers into international financial markets, which in turn may create or magnify risks to financial stability in other countries. To contain the side effects of monetary policy for financial stability at both the national and international levels it is necessary to set up strong macroprudential policy frameworks across all relevant jurisdictions. But although in principle international coordination of countercyclical monetary and macroprudential policies may help, in practice it may compound the difficulties highlighted earlier with regard to maintaining cooperative agreements – the incentive to renege, the divergence in views regarding how the world works and how policies affect it, and so on.

## Promoting international macroprudential policy coordination: regulatory standards and reciprocity principles

As discussed earlier, in recent years increased interconnectedness of financial institutions and markets, and more highly correlated financial risks, have intensified the strength and speed of cross-border spillovers. At the same time, there has been increased recognition that differences in national macroprudential policy regimes across countries can themselves be a source of international spillovers. In particular, by triggering cross-border regulatory arbitrage, these differences may lead to large swings in capital flows and magnify the international transmission of real and financial shocks. In turn, this may exacerbate financial risks locally if credit is already growing rapidly in recipient countries. When global financial intermediaries can evade policy actions taken by national authorities, and financial cycles are not well synchronized across countries, the combination of national macroprudential policies may be sub-optimal from the perspective of the world economy – even when each country’s macroprudential policy is optimal at the national level.

In this section we discuss the practical aspects of promoting cross-country coordination of macroprudential policies in an interconnected world. We consider macroprudential policy coordination in its time-series dimension, through reciprocity agreements at two levels: minimum regulatory standards and countercyclical responses. We also discuss coordination with respect to the imposition of capital surcharges for systemically important banks, which relates to the cross-sectional dimension of macroprudential regulation. This analysis sets the stage for a discussion of a more specific approach to promote coordination in the next section.

### Minimum regulatory standards^50^

As noted earlier, national macroprudential policies that are designed to contain risks associated with a rapid expansion of domestic credit can be subject to leakages from an increase in cross-border borrowing, which in turn may weaken their effects. In addition, during a crisis or in its immediate aftermath, a protectionist national financial policy response may favor local banks. When that occurs, fragmentation increases, with the best example being Europe where the intertwined problems of banks and sovereign risks culminated in the 2010–12 Eurozone debt crisis (see Baldwin and Giavazzi (2015)). Global coordination may help to avoid these outcomes.

The first example of such coordination is through an internationally agreed structural minimum standard on capital requirements to guard against regulatory arbitrage. Indeed, the goal of the first Basel Accord, introduced in 1988, was to harmonize capital regulation across jurisdictions. Reciprocity was aimed at ensuring that the same standard was imposed on all relevant credit exposures to borrowers in a given country – regardless of whether credit is provided by domestic or foreign entities.

The Basel framework evolved in accordance with the perception and measurement of risks, as well as their international transmission. One direction taken, in 1996, was to consider using banks’ internal models for regulatory capital requirements for market risk. Under Basel II, introduced in 2004, banks got the option of using their own credit risk estimates under the internal ratings-based approach (IRB). The goal was to reduce the scope for arbitrage and provide banks with incentives for improved risk measurement and management. However, the GFC laid bare the fact that in many countries, banks had excessive leverage, inadequate and low-quality capital positions, and engaged in excessively risky activities. In effect, the financial system de facto relied too heavily on risk-weighted capital ratios to assess both individual and systemic risks. As documented in numerous studies, the inadequate ability to assess financial risks in a favorable macroeconomic environment was a contributing factor to the GFC (see, for instance, Thakor (2015)).

The Basel III framework, introduced in 2010 and updated in 2011, aimed to address weaknesses both in banks’ risk management and in other dimensions of the regulatory framework that were exposed by the GFC. Key shortcomings included insufficient loss-absorbing capital, unsustainable leverage and inadequate liquidity buffers. In addition, too little attention was paid to the systemic risks looming in the financial system as a whole. In response, the Basel III framework set significantly higher loss-absorption requirements and puts greater emphasis on capital quality, while broadening the coverage of bank risks (see Basel Committee on Banking Supervision (2011)). Important new aspects of the framework include a leverage ratio requirement, capital overlays targeting various sources of systemic risk (including the countercyclical capital buffer, discussed next), and a set of internationally harmonized standards limiting liquidity and maturity transformation, such as the Liquidity Coverage Ratio and the Net Stable Funding Ratio (see Basel Committee on Banking Supervision (2013)).

Furthermore, supplementary requirements for global systemically important banks (G-SIBs) have been developed to strengthen the resilience of these banks and contain any adverse impact in the event of failure.^51^ These requirements are aimed at reducing too-big-to-fail risks through a variety of measures designed to help internalize the resulting externalities. They include specific capital surcharges but also structural requirements (such as the development of “living wills”) to facilitate the resolution of a G-SIB. These surcharges seek to offset the additional systemic risk associated with the large size, complexity and interconnectedness of G-SIBs (see next subsection). In addition, other measures, such as the new Total Loss Absorbing Capacity (TLAC) requirement, ensure sufficient buffers of resources before bank capital is exhausted.^52^

### Global systemically important banks: capital surcharges

The crisis demonstrated key gaps in the framework for the resolution of systemic financial institutions, or SIFIs, including those with extensive cross-border operations, G-SIFIs, and especially G-SIBs, referred to earlier.^53^ Recognition of the unique nature of G-SIFIs – with global activities, but regulatory and resolution authorities largely circumscribed by national boundaries – has led in recent years to international cooperation in designing a consistent framework covering the resolution of these institutions. The key motivation is that the failure of one G-SIFI can send contagious shockwaves across national borders and lead to a squeezing (or even a seizing up) of liquidity in key financial markets, with adverse effects on the provision of credit to the real economy – even in countries where banks were not exposed to the underlying risks.

Therefore, the Basel Committee and the Financial Stability Board (FSB) have developed a framework to globally assess newly required capital surcharges and their application to G-SIBs. Furthermore, the proposed framework to deal with banks that are systemically important from a *domestic* perspective (which are more numerous than G-SIBs) sets out principles that govern the interaction between the assessment and actions of a bank’s host supervisor and those of its home supervisor. Indeed, the agreements are supplemented by guidelines that reflect some discretion for national authorities to assess capital surcharges for domestic SIBs and seek some international consistency of approach.

### Reciprocity and countercyclical capital buffers

The Basel Committee on Banking Supervision (BCBS) established the principle of *jurisdictional reciprocity* in the context of the use of countercyclical capital buffers.^54^ Under this principle, foreign supervisors must apply (at least) the same additional capital buffers imposed by the host supervisor to their banks’ lending to the host country. The goal is to ensure that all banks operate on a level playing field when lending to entities in the host country.^55^ Moreover, the principle aims not only to address the issue of regulatory arbitrage but also to help whenever credit exposures are large, and hence systemic with respect to the host country, but small and hence of little significance in relation to the lending institution’s portfolio – a fairly common situation, as noted earlier, given the size of internationally active banks.^56^

The reciprocity clause built into global rules on cyclically-varying capital buffers may help alleviate the problem of leakages and tackle the inaction bias, alluded to earlier, inherent in macroprudential policy. It also provides an important first step toward an international coordination regime for countercyclical macroprudential regulation. However, it also faces some limitations (see Viñals and Nier (2014)). First, rather than helping alleviate the problem of leakages, in practice it may create (if only temporarily) incentives for banks to increase their exposures to countries with no (or a relatively smaller) capital buffer requirement in place, and conversely to reduce their exposures to countries that have imposed a relatively larger buffer. There would therefore be a greater concentration of risky activities in relatively lightly regulated jurisdictions. However, given that increases in countercyclical capital buffers are (by definition) temporary, these distortions may not materialize quickly enough to become a source of concern. Moreover, these migration effects could be addressed in part by high minimum standards (as discussed earlier), as well as supplementary agreements – including additional charges for domestic SIBs and the minimum internal TLAC requirements to be applied to each resolution entity within each of these institutions, as discussed by the Financial Stability Board (2014) – although this may not cover all jurisdictions.

Second, the reciprocity principle only applies to countercyclical capital buffers and not to the entire range of macroprudential tools, including sector-targeted risk-weighted measures (such as loan-to-value or debt-to-income ratios, or cyclically-adjusted loan loss provisions) that regulators may have at their disposal and may consider when facing increased risks of financial instability in the time-series dimension. Thus, these other macroprudential tools may continue to be subject to leakages caused by cross-border financial transactions. At the same time, however, it should be recognized that coordination of countercyclical responses in terms of these instruments (for instance, loan-to-value ratios) may not be feasible, due to a lack of synchronization across countries (in real estate markets in particular) or because of idiosyncratic differences in legislation.

Third, the principle applies only to banks; in countries where the shadow financial system accounts for a growing share of domestic credit, a narrow regulatory perimeter affects the ability to mitigate financial risks. Fourth, in a world consisting of advanced economies with highly developed financial systems and developing economies (including most of the SMICs) with less sophisticated financial markets and more limited resources to oversee their financial institutions, some countries in the latter group may still be in the process of building up their macroprudential regime. They may not have the same supervisory capacity as advanced economies – hampering therefore their ability to reciprocate. Moreover, for those countries where the exposure to the host country is a small share of the total exposures of the home country financial system, or when domestic credit growth (a common trigger for countercyclical capital buffers, as discussed, for instance, by Drehmann and Tsatsaronis (2014)) is moderate, there may be little urgency from the national perspective to impose constraints on cross-border exposures – even though these exposures may constitute a sizeable share of the total credit provided in the host country. There is a risk then that those countries wishing to tighten macroprudential regulation, but whose efforts are hampered by increases in cross-border credit, will resort to more distortive measures, such as (as mentioned earlier) the imposition of capital controls to impede capital inflows – with possible adverse deflection effects on other countries.^57^ To prevent such outcomes, international coordination should not be limited to a narrow set of instruments.

## Strengthening the framework for international coordination of macroprudential policies

As documented previously a number of recent contributions have established that under some circumstances potentially significant gains can be achieved for the world economy if macroprudential policies are coordinated across countries, compared to noncooperative policies. Indeed, the very use of macroprudential policies at the individual country level may be ineffective in a financially integrated world economy with global banks, as a result of financial spillovers and cross-border regulatory leakages. Moreover, we have also argued that in the case of the financial relations between major advanced economies and SMICs, the potential disruptive effects of spillbacks from the latter group to the former can be large enough to make a case for prevention through *ex ante* coordination in the very self-interest of advanced economies.

At the same time, however, international coordination of countercyclical macroprudential policies has been viewed by some as being somewhat unrealistic and unlikely to occur in practice, considering the narrow nature of national mandates bestowed to central banks, regulators and supervisors, incentives to deviate from agreed policies, and uncertainty about the magnitude and sign of spillovers and spillbacks. In addition, as noted earlier, quantifying the gains from coordination depends heavily on the type of models and metric used (policy loss functions or household utility) in estimating the difference between cooperative and noncooperative equilibria.^58^ The issue then is the following: if there is an analytical case for coordination but with parts still missing – especially with respect to quantifying the gains from coordination – how can we promote a pragmatic approach to international macroprudential policy coordination between countries that may potentially benefit the most from it, namely, major advanced economies and SMICs, given their increased degree of trade and financial interconnectedness?

### Coordinating about coordination

To promote macroprudential policy coordination among major advanced economies and SMICs, a sensible approach would be to use the existing international cooperative arrangement, involving the IMF, the BIS, and the FSB, to develop the following agenda:

1. Continue the statistical effort through which information about the types, timing and circumstances of usage of macroprudential instruments is currently collected, formatted and disseminated (see IMF-FSB-BIS (2016) and Alam et al. (2019)); in particular, the current datasets need to incorporate more granular information about the nature (structural or countercyclical), direction (tightening or loosening) and intensity (vis-à-vis some initial conditions) in the usage of the range of available macroprudential instruments as well as their effectiveness in affecting the financial cycle. International cross-border data on capital flows by agent and nature of transaction are important to assess the benefits from international coordination of macroprudential policies.^59^
2. Explore further the evidence on financial cross-border spillovers, dwelling on the literature on the topic (see, for instance, Buch and Goldberg (2017), Buch et al. (2019), and Cappelletti et al. (2020)) to improve existing models of spillovers, their underlying methodology, and better understand policy responses.
3. Improve the measurement of the national and cross-border effects of the implementation of macroprudential tools. At the national level, and as noted earlier, the evidence on the benefits of macroprudential policies is still mixed and could be improved with better data. At the international level, as also noted earlier, there is evidence that cross-border financial spillovers and spillbacks have increased in magnitude in recent years – and so have the potentially negative externalities associated with them, especially in countries where financial systems tend to be highly procyclical. By implication, international coordination of macroprudential policies can lower the risk of a global financial crisis or regulatory wars – but the channels through which this may occur need to be better understood. This requires improving modelling tools and their ability to take into account the cross-country general equilibrium effects associated with real and financial spillovers.
4. Accumulate further analytical and empirical evidence regarding the potential gains of macroprudential policy coordination, in both its structural and countercyclical dimensions. In that particular aspect, the BIS (perhaps in a collaborative effort with the FSB) could further strengthen its current research effort in order to produce a regular and comprehensive assessment on international macroprudential policy coordination encompassing statistical, empirical and analytical contributions.
5. Develop better indicators and models to assess systemic risk both within and outside the banking system, especially regarding the activities of shadow banks. Because financial stability is a broad concept with several dimensions (as noted earlier), including a complex relationship between national and international levels, no common metric exists and it may not be possible to establish a well-accepted one (comparable to the role that, for instance, the consumer price index plays in an inflation targeting regime). More analytical and applied research is required, not least to better identify what kind of data are needed, when and how these data should be collected, and what type of modelling framework is warranted.^60^

More generally, international coordination of macroprudential policies needs to be built not only on shared information, but also on shared analysis. Various sharing mechanisms already exist: they include IMF surveillance reports and Financial Sector Assessment Program (FSAP) assessments, FSB peer reviews, and bi-monthly meetings of senior central bank officials at the BIS. The BIS also regularly conducts quantitative impact studies (QIS) for the Basel Committee, with the goal of assessing the effect of implementing specific pieces of financial sector regulation. An example of this on-going approach occurred with the QIS on the implementation of the Basel III countercyclical capital buffer.^61^ But more needs to be done with tools that provide a more comprehensive explicit modelling of national and international transmission of macroprudential policies.

The capacity to develop a modelling framework with some common core elements is also important to provide legitimate advice. As noted earlier, the lack of consensus on the direction and magnitude of spillovers, and the impact of policies to mitigate them, can undermine international cooperation – especially with respect to countercyclical responses, which often require timely decisions.^62^ An analytical effort to develop some common model – dwelling, for instance, on multi-country models already in use in several international institutions – to provide robust evidence on the gains from coordinating policies may not, of course, change current mindsets and assuage doubts overnight, but it may help to confront points of view and discuss why countries may disagree. In addition, there is a need to assess the welfare losses resulting from the lack of coordination, which may take the form of financial protectionist measures, such as capital controls. Such an outcome could indeed emerge in a world of excessive volatility in capital flows and unwillingness by major advanced economies to engage in policy cooperation, leaving major middle-income countries with no other option but to impose restrictions on capital movements.

A common work agenda in these directions could promote a better understanding of the need for macroprudential policy coordination for two main reasons. First, as mentioned above, there is a need to effectively and credibly estimate, as carefully as possible, the cross-border effects associated with the implementation of macroprudential regulation, differentiated by types of instruments. In doing so, it is essential to avoid capture of these calculations by vested national interests, and this can be better achieved through the cooperative work of international institutions that are considered credible and legitimate actors. The BIS, FSB and IMF are already active in this field and have conducted joint analyses in the past (see IMF-FSB-BIS (2016)). Most of the countries (including all the SMICs) that would get to benefit more directly from increased coordination are members of these forums.

Some problems will surely remain: what if there is no agreement on the very definition of financial stability, and a common modelling framework or yardstick to measure the magnitude of financial spillovers and spillbacks, the gains from macroprudential policy coordination, or the appropriate policy responses? What if participants in discussion forums do not reach common ground on these issues? Sharp and well publicized disagreements could have adverse effects on credibility, which in turn could undermine the legitimacy of the proposed work program and its ability to influence policy choices. At the same time, this scenario is not new; indeed, it has been a perennial issue confronting international cooperation on a broad range of matters. In fact, this is an argument that also favors a *tripartite* approach in some of the aspects of the proposed work agenda. It is easier not to pay attention to one individual international institution. For instance, the IMF’s process of multilateral surveillance between 2004 and 2007, designed to produce concrete actions to reduce global imbalances, was largely unsuccessful (see Butler (2012)), possibly because the diagnostic, irrespective of its accuracy, was not as widely shared as one would have hoped. But it would be more difficult not to listen to a set of robust empirical and analytical results coming from a *group* of well-established institutions, which together represent best practices and policy advice on promoting macroeconomic and financial stability. More generally, if the goal is to promote closer coordination between countries, starting with sound analytical work carried out within the international institutions with a direct stake in the stability of the international financial system would be an important step forward.

## Summary and concluding remarks

The purpose of this paper has been to discuss the scope for international macroprudential policy coordination in a financially interconnected world economy, and assess how such coordination can be promoted in practice. Several key lessons have emerged from our analysis. First, with the advance in global financial integration over the last three decades, the transmission of shocks has become a two-way street – from advanced economies to the rest of the world, but also, and increasingly, from a group of large middle-income countries, which we refer to as SMICs, to the rest of the world, including major advanced economies. These increased spillbacks have strengthened incentives for advanced economies to internalize the impact of their policies on these countries, and the rest of the world in general. Although stronger spillovers and spillbacks are not in and of themselves an argument for greater policy coordination between these economies, the fact that they may exacerbate financial risks – especially when countries are in different phases of their economic and financial cycles – and threaten global financial stability is.

Second, the disconnect between the global scope of financial markets and the national scope of financial regulation has become increasingly apparent, through leakages and cross-border arbitrage, especially through global banks. In fact, what we have learned from the financial trilemma is that with open capital markets it has become increasingly difficult to maintain domestic financial stability without enhancing cross-border macroprudential policy coordination, at least in its structural dimension. Addressing the cyclical risks created by international regulatory arbitrage also requires coordination.

Third, divergent policies and policy preferences create additional dimensions to global financial risks. In the absence of a centralized macroprudential authority, coordination needs to rely on an international macroprudential regime that promotes global welfare. To mitigate the obstacles that national interests can create, global institutions have an important role to play. Fourth, significant gaps remain in the evidence on regulatory spillovers and arbitrage, and the role of the macroprudential regime in the cross-border transmission of financial shocks. In addition, research on the potential gains associated with multilateral coordination of macroprudential policies remains limited. This may be due in part to the natural or instinctive focus of national authorities on their own country’s objectives, or to greater priority on policy coordination within countries – an important ongoing debate in the context of monetary and macroprudential policies, given that these policies tend to operate through similar channels. This “inward” focus may itself be due to inadequate awareness of the benefits of multilateralism for achieving national objectives, which therefore makes further research on these benefits all the more important.

This assessment suggests that, in a financially integrated world, international coordination of macroprudential policies may not only be valuable, but also essential, for macroprudential instruments to be effective at the national level. A first step toward coordination was taken with Basel III’s principle of jurisdictional reciprocity for countercyclical capital buffers, but this principle needs to be extended to a larger array of macroprudential instruments. Further empirical and analytical work (including by major financial institutions) on the benefits of international macroprudential policy coordination could play a significant role in promoting greater awareness of the potential gains associated with global financial stability. This work agenda should involve a research component focused on measuring the gains from coordination and improving data on cross-border financial flows intermediated by various entities (banks, investment funds, and large institutional investors), as well as improving capacity for systemic risk monitoring.

## Footnotes

1 See Lane and Milesi-Ferretti (2017). As noted later, this slowdown had only a limited impact on the degree of banking globalization.

2 See Agénor (2012) for a review of the literature on the benefits and costs of international financial integration. Obstfeld (2015) provides a broader perspective.

3 See Bagliano and Morana (2012), Bauer and Neely (2014), Fratzscher et al. (2014), Aizenman et al. (2016), Tillmann (2016), MacDonald (2017), Tillmann et al. (2019), Ca 'Zorzi et al. (2020), and Ahmed et al. (2021) for a discussion of these spillover effects. Some of these contributions focus on the effects of the Federal Reserve’s large-scale asset purchase (LSAP) program.

4 Broader reasons for operating a managed float in many middle-income countries include the impact of currency fluctuations on domestic inflation, the domestic-currency value of foreign liabilities, and competitiveness. See Agénor and Pereira da Silva (2019*b*) for a discussion.

5 Financial market spillovers to advanced economies from the rest of the world are now broadly referred to as spillback effects; a more precise definition is provided later on.

6 Macroprudential policy is usually defined as actions taken by regulatory authorities in their own jurisdiction aimed at promoting financial stability and mitigating systemic risks to the financial system (see Committee on the Global Financial System (2010)). These actions use a set of instruments that reduce the vulnerabilities (leverage, market risk or interconnectedness) of the financial system by imposing specific rules or restrictions on the balance sheets of lenders, lending contracts, other nonbank financial agents, and the market infrastructure itself.

7 Korinek (2017) derived a welfare theorem for open economies which shows that the conditions that need to be violated to generate Pareto inefficiency under an uncooperative equilibrium and create scope for cooperation are unlikely to hold in practice. However, the premise of his analysis is that the goal of cooperation is to restore competitive behavior, rather than mitigating financial stability risks to the global economy.

8 The term “emerging markets” lumps together a fairly disparate group of economies – large and small countries (for instance, China and the Czech Republic), rich and poor countries, manufacturing and commodity-based exporters, and countries with large external deficits and large surpluses. For many substantive issues, including the one addressed in this paper, this amalgam does not help to bring to the fore some of the relevant characteristics. In addition, international organizations (such as the International Monetary Fund and the United Nations) and private institutions involved in the publication of financial indices (such as MSCI, JP Morgan Chase and FTSE) use a clutter of conflicting criteria to categorize countries they include in the “emerging markets” group. This creates confusion and inconsistencies when making comparisons across measures or over time. Even accepting prevailing classifications, it is often unclear why one country is labelled “emerging” while another is labelled “developed.” For instance, prior to the COVID-19 pandemic, Chile had a larger economy, a bigger population, a lower level of public debt and lower unemployment than Portugal but was classified as emerging, whereas Portugal was included in the “advanced economies” category. Similarly, according to World Bank data, on a per-capita income basis, South Korea was wealthier than several advanced economies (including Portugal and Spain) in 2019 but was still considered as “emerging.”

9 According to the World Bank’s classification, India and Indonesia are lower middle-income countries, whereas the others are all upper middle-income countries. See https://datahelpdesk.worldbank.org/knowledgebase/articles/906519-world-bank-country-and-lending-groups.

10 International coordination between large economies and smaller economies may be less probable if the potential gains are likely to be small (Kincaid and Watson (2016)). However, small economies may indirectly benefit from the coordination between major advanced economies and SMICs, to the extent that it promotes global financial stability. In addition, coordination between large and smaller countries on the *structural* dimension of macroprudential policy – through implementation of the Basel III standards or (as discussed later) extensions of them – is of course desirable and potentially beneficial for the global economy as a whole.

11 After a first draft of this paper was completed, we became aware of a contribution by Huidrom et al. (2017), which takes a position related to ours – they suggest focusing on the largest emerging market economies or EM7 (our group of SMICs minus South Africa) because of their importance in terms of global output and their potential for large cross-border spillover effects. However, their focus is on *growth* spillovers, whereas our focus is on financial spillovers. Moreover, they do not discuss the benefit of their proposed grouping in the context of policy coordination issues.

12 Macroeconomic models that account for financial frictions, as well as a range of interactions between the real and financial sectors, are now commonly used in academic circles, central banks and research institutions to study the benefits of macroprudential regulation, independently and in combination with monetary policy, in both closed and open economies. For a thorough discussion, see Agénor (2020).

13 Some recent discussion has also focused on cross-border *fiscal* spillovers. The European Commission (2014), for instance, estimated that an increase in public investment in euro area countries with fiscal space would generate significant positive spillovers to the other members. See also International Monetary Fund (2020).

14 When discussing and measuring the global transmission of financial shocks, distinguishing between *interdependence*, which refers to the correlation across financial markets during “normal” states of the world, and *contagion*, is essential. While there is no full agreement on the definition of contagion, most studies define it as the spillover effects taking place above and beyond the linkages explained by fundamentals, or as the extreme amplification of spillover effects (see Forbes (2012)).

15 For instance, a decision by global mutual funds to sell investments in multiple countries in response to losses in one or more countries, or because of fund withdrawals by their own investors, is usually referred to as the portfolio channel of contagion. This channel has gained in importance as a source of financial spillovers from large developing economies to equity markets in recent years, in line with the increase in asset allocation to these countries. Nevertheless, as discussed later, the impact of the portfolio channel emanating from advanced economies remains significantly larger.

16 As documented by the International Monetary Fund (2016b), financial spillovers from China to regional markets in Asia – in particular, equity and foreign exchange markets – have not only risen since the GFC but are also stronger for those economies with closer trade linkages with that country.

17 The GFC had only a limited impact on this trend, even though there was some post-crisis retrenchment of major global and non-major European banks operations in the aggregate. See Claessens and van Horen (2014), Cerutti and Zhou (2017) and McCauley et al. (2017) for a discussion of the evidence.

18 See Cecchetti and Tucker (2016) for a more detailed discussion of these transmission channels, and Cetorelli and Goldberg (2012), and Buch and Goldberg (2017). Krugman (2008) discussed a related idea to the credit channel, which emphasizes how interconnections in financial markets may give rise to an international finance multiplier.

19 Financial institutions, including their branches, are generally supervised on a consolidated basis by their home supervisor. Host countries supervise their domestic financial institutions, which include subsidiaries of foreign financial institutions. Thus, macroprudential tools applied by a host country would not apply to branches located in the host country.

20 Other channels that have been explored in the empirical literature include uncertainty (Chuliá et al. (2017)), confidence (Dées and Guntner (2014) and Kamber et al. (2016)), and the degree of financial integration itself (Pyun and Jiyoun (2016)). Given the focus of this paper, we refrain from a more detailed discussion.

21 Other studies worthy of note include those by Fratzscher et al. (2014) and Mishra et al. (2014), whose focus is on correlations in equity and foreign exchange returns, and the related literature by De Bruyckere et al. (2013) and Lucas et al. (2014); those by Favero (2013), Alter and Beyer (2014), and Disyatat and Rungcharoenkitkul (2017), whose focus is on correlations in returns on sovereign bonds; and those by Beltratti and Morana (2010) and Hirata et al. (2012), whose focus is on correlations in house prices.

22 The IMF studies also found that news about China’s economic growth had a rising, and economically significant, impact on world financial markets; in particular, the impact of unexpected growth shocks from China on global equity prices almost quadrupled in the aftermath of the GFC. For instance, the sharp sell-offs in global stock markets that occurred in August 2015 and January 2016 appeared to have been significantly related to news of an activity slowdown in China. By contrast, changes in Chinese asset prices continued to have little *systematic* effect on asset prices elsewhere – even though *specific* episodes of spillovers to global equity markets can be identified, as was the case in the aftermath of the sharp drop in Chinese equity markets in August 2016, which followed the announcement of a change in the country’s exchange rate regime. Fang et al. (2021) also found that fluctuations on Chinese financial markets had a growing impact on global financial markets over the period 2000–18, especially during periods of turbulence. As China continues to integrate into the global financial system, purely financial spillovers from that country are likely to grow significantly.

23 This sample selection is important because global financial and economic integration is a key driver of monetary spillovers. It determines the strength of investor arbitrage by tying bond market rates to those prevailing in the core economies. It also affects the degree to which policymakers might be concerned about capital flows and exchange rate volatility – concerns that would induce them to factor US interest rates into their monetary policy decisions.

24 See also Buitron and Vesperoni (2015) and Chatterjee (2016) for similar results. A related study by Chuliá et al. (2017) analyzed the impact of US policy and US equity market uncertainties on stock returns abroad.

25 See Buch and Goldberg (2017) for a summary of the evidence. Note that although our interest in this paper is on cross-border *macro*prudential policy leakages (as noted previously), we discuss in this subsection the broader evidence on regulatory leakages. The reason is that, in practice the same prudential instrument (say, capital requirements) can be changed for either *micro* or *macro*prudential motives, depending on the *intent* of the regulator. This is actually the case for most prudential instruments in the time-series dimension. Because intents are generally not observed, there is prima facie a serious identification problem for empirical studies aimed at isolating the effects of macroprudential policy measures – except for those focusing on specific events, where the policy motivation can be clearly established. We therefore take a broader perspective in our review of the cross-border effects of regulatory policy changes.

26 See Georgiadis (2017) for a discussion of indirect (as opposed to direct, or bilateral) higher-order spillovers and spillbacks, and how they relate to the degree of trade and financial integration.

27 See Galati and Moessner (2013) and Claessens (2015). Because financial *in*stability is easier to identify than financial stability, financial stability has sometimes been defined as *the absence* of a systemic crisis. But this definition is not helpful from an operational standpoint because, before a financial crisis occurs, the financial system may still be in a highly vulnerable state.

28 The evidence on the link between (excessive) credit growth and financial crises is strong and covers both advanced and developing economies. Fielding and Rewilak (2015), for instance, found that credit booms increase the likelihood of a banking crisis in relatively fragile financial systems, that is, those characterized by a poor financial performance of banks. Other contributions include Aikman et al. (2015) and Aldasoro et al. (2018). Taylor (2015) and Agénor and Pereira da Silva (2019*b*) provide a more comprehensive list of references.

29 See Cerutti et al. (2017a), Akinci and Olmstead-Rumsey (2018), and Borio et al. (2021) on the effectiveness of macroprudential policies. On the issue of complementarity or substitutability between macroprudential and monetary policies, see Adrian and Nellie Liang (2018), Agénor and Pereira da Silva (2019*b*, 2021), and Agénor and Flamini (2021), as well as the references therein.

30 In the policy world, cooperation is typically taken to mean collaboration via sharing of information, discussion of common issues and one-off emergency responses, especially during periods of financial stress. Coordination refers to policy actions formally agreed and taken by groups of policymakers – possibly including multilateral institutions – aimed at achieving beneficial outcomes for the international community as a whole. In what follows we maintain this distinction for clarity.

31 Under a fixed exchange rate regime or a managed float, with limited scope for conducting an independent monetary policy, macroprudential instruments may be directed not only toward mitigating financial risks but also toward achieving macroeconomic objectives – although, in the latter case, benefits to financial stability may also result. This has often been the case in Latin America; see Agénor and Pereira da Silva (2019*b*) for a discussion.

32 Obstfeld (2015) stressed that areas of cross-border coordination relate not only to financial regulation, but also to clear rules of the game for capital controls and enhanced facilities for international liquidity support in key currencies to counteract the downside effects of excessive reserve accumulation.

33 Traditional arguments for international coordination of banking regulation are the need to maintain a level-playing field for banking competition and avoid regulatory races to the bottom. These arguments also apply to the macroprudential dimension of banking regulation.

34 Although our focus in this discussion remains on international coordination, strengthening national mandates for financial regulators to ensure independence and legitimacy to take appropriate macroprudential policy decisions are also important from that perspective.

35 See VanHoose (2016) for a survey of that literature.

36 In addition, unlike Dell’Ariccia and Marquez (2006), who explored differences between countries only in terms of the regulators’ exogenously specified preferences, Kara (2016) considered asymmetries that are due to endogenous structural differences across countries, such as differences in productive technologies. The implications of these structural asymmetries for the feasibility of coordination are also examined, with mixed results.

37 See Ostry and Ghosh (2013, 2016), who argued that international negotiations may actually harden differences in perceptions about the way the world works.

38 See VanHoose (2016) for a formal example, with a *race-to-the-bottom* outcome in terms of bank regulation.

39 Taylor (2013) and Frankel (2016) provided a critical review of the early academic literature on the gains (or lack thereof) from international monetary policy coordination.

40 However, this latter point depends on the structure of the model; as shown by Obstfeld and Rogoff (2002), the case for coordination may be the weakest precisely when goods market integration is high.

41 Benes et al. (2016), for instance, argued that the nonlinearities associated with the financial system (namely, the convex relationship between lending risk premia and loan-to-value ratios), rather than goods market interactions, are one of the key reasons why internationally coordinated policies may lead to sizeable output spillovers. However, they do not evaluate the gains from macroprudential coordination per se. In addition, it should be pointed out that Banerjee et al. (2016) did not find large gains associated with monetary policy coordination in a model with financial frictions. Yet, it is also worth noting that their analysis assumes that these frictions are symmetric across countries – an assumption that is not warranted when the focus is on coordination between advanced economies and large developing countries, as is the case in the literature discussed next.

42 Establishing that the macroprudential regime matters in the transmission of shocks across countries, and that macroprudential instruments are effective in terms of mitigating financial risks at the national level, can be viewed as preconditions for making a case for international macroprudential policy coordination.

43 Aizenman et al. (2020) found that tighter macroprudential policies in periphery countries helps them to regain monetary independence from core countries – the more so when their financial markets are relatively closed.

44 Recent contributions based on multi-country models of the world economy with extensive macro-financial linkages include Benes et al. (2016), Dieppe et al. (2017), and Vitek (2017).

45 A related contribution, by Agénor et al. (2021a, 2021b), focuses on the gains from coordination associated with Basel III’s principle of jurisdictional reciprocity, which is discussed later on.

46 See Bank for International Settlements (2016, chap. 4), Adrian and Liang (2018), Agénor and Flamini (2021), and Agénor and Pereira da Silva (2021), for a discussion. For a dissenting view, see Svensson (2016), who argues that with less effective macroprudential policies, using monetary policy entails too high a sacrifice ratio. One caveat to all this literature, however, is that the effectiveness and calibration of macroprudential instruments remain insufficiently understood, compared to monetary policy.

47 Bruno et al. (2017), for instance, found that macroprudential policies tend to be more successful when they complement monetary policy in Asian economies, by reinforcing monetary tightening, rather than when they act in the opposite direction. Gambacorta and Murcia (2016) found that macroprudential policies that are used as complements of monetary policy have larger negative effects on credit growth than other types of measure. Moreover, the degree of complementarity between monetary and macroprudential policies depends on the type of policies implemented: policies with countercyclical objectives are more likely to exhibit complementarity with monetary policy than policies (such as the setting of capital ratios) that are more structural in nature.

48 The issue of coordination of macroeconomic policies, at both the domestic and international levels, also arises for other combinations of instruments. Gaspar et al. (2016), for instance, discussed the scope for international coordination of monetary and fiscal policies to sustain global output growth.

49 The creation of US dollar liquidity swap lines in December 2007, which involved central banks in both advanced and middle-income countries, is a good example of monetary policy cooperation at a time of crisis – a sort of “Global Lender of Last Resort,” as pointed out to us by Hiroshi Nakaso. In October 2013, these swap lines were converted to standing arrangements. The agreement among G-7 partners in February 2013 on a “Currency War Ceasefire” represents the most substantive example of international economic policy coordination in the last few years (see Frankel (2016)). However, the agreement made no explicit reference to monetary policy and did not mention what sanctions could be imposed, if any, to enforce it.

50 This section is based in part on Caruana (2016).

51 The Financial Stability Board and the Basel Committee on Banking Supervision defined (in 2012) and regularly publish the results of a methodology to identify G-SIBs that includes cross-jurisdiction activity, asset size and interconnectedness. See www.bis.org/bcbs/gsib/.

52 Another issue for the regulatory agenda is the comparability of variation in risk-weighted assets (RWA) between banks using their internal models (IRB) with those using a standardized approach (SA). Ideally, the same risks should require the same level of risk capital and also similar risk weights. Therefore, the issue is how to separate a natural variation in model outcomes (such as differences in portfolios and model parameters) from variation arising from banks’ tactical calibration choices (“gaming”), sometimes compounded by differences in supervisory approaches across jurisdictions. A number of studies conducted by several experts and the FSB suggest that “gaming” has contributed to material variations in reported RWAs across banks, reducing the true risk sensitivity of the RWAs and undermining comparability across banks and the enforcement of market discipline. To narrow IRB banks’ incentives to artificially “game” their risk estimates may require measures to limit their capacity to pick model parameters (such as minimum risk weight floors) and greater scrutiny with regard to IRB model approval criteria.

53 SIFIs encompass banks, insurance companies, and other financial institutions which, in the event of financial stress or failure, can trigger a global and potentially severe financial crisis.

54 Fundamentally, the countercyclical capital buffer is intended to counterbalance the procyclical behavior of banks by building up buffers in good times that can absorb losses in times of stress. It is a prudential instrument calibrated to achieve a macroprudential objective. Critically, the level of the buffer depends on the state of the financial cycle in a given jurisdiction. The framework allows for a large degree of judgment and tailoring to local circumstances.

55 More precisely, under these provisions the home country of an international bank must impose a capital buffer that is a weighted average of the capital requirements in the host countries where the bank operates, with weights determined by the share of the bank’s exposure in different countries. For instance, a bank that has half of its exposure in its home country, and half of it in another country will face capital requirements equal to the average of the two countries’ required capital ratios – subject to the provision that the requirements be no lower than those imposed by the home country on domestic banks. Reciprocity becomes binding when some countries decide to impose stronger requirements than the minimum recommended in the current Basel III regime.

56 Authorities in the European Union (EU) have developed a similar, voluntary approach aimed at all measures targeting exposures. Guidance is also provided to EU countries on how to treat exposures to third countries that fail to take macroprudential measures (see European Systemic Risk Board (2014) and Financial Stability Committee (2020)). Cantone et al. (2019) provided evidence for the EU supporting the introduction of mandatory reciprocity arrangements for sectoral capital buffers to ensure a level playing field and pre-empt leakages across borders.

57 Forbes et al. (2016) found that Brazil’s taxes on fixed income and equity aimed at stemming capital inflows into these markets diverted capital flows into other Latin American countries. Similar evidence for a broader group of countries is provided by Ghosh et al. (2014) and Giordani et al. (2017). As noted by Ostry et al. (2012), to the extent that capital controls help countries to sustain undervalued currencies, they may also contribute to global imbalances.

58 Moreover, it is clear from the past and on-going difficulties of the Basel process (for instance, its current unanimous decision rule with a smaller insider group of primus inter pares advanced economies), that political economy considerations often play a role when countries complain about other countries’ policy actions. As noted by Frankel (2016), these concerns are sometimes put forward to avoid working on one’s necessary adjustments and reforms, and thus avoid addressing domestic distortions and disagreements among special interest groups.

59 At present most efforts to measure spillover risks look at measuring stock market risk, asset price bubbles, credit growth, interest rates, other financial variables and real variables. The BIS and IMF have taken the lead in monitoring cross-border spillovers and global liquidity through their consultation and coordination role with national central banks and governments respectively (see Eickmeier et al. (2013)). This macro-finance perspective relies on high frequency data from financial markets and attempts to capture macroeconomic imbalances and asset price bubbles.

60 A promising new direction is the measure of financial stability based on the probability distribution of future GDP growth developed by the International Monetary Fund (2017). However, further validation tests are needed.

61 See https://www.bis.org/bcbs/qis/.

62 This view is consistent with Bayoumi (2014), who emphasized the need for greater consensus on estimated spillovers to promote international cooperation, and Eichengreen (2014), who suggested (based on historical evidence) that international economic policy coordination tends to be more successful when it involves broad agreement among experts on technical matters.
